# RECQL4 promotes the malignant progression of lung adenocarcinoma through the YBX1/G3BP1-mediated NF-κB signaling pathway

**DOI:** 10.1038/s41420-025-02849-3

**Published:** 2026-01-09

**Authors:** Rongyang Li, Wenhao Yu, Dingxin Wang, Luyuan Ma, Zhanpeng Tang, Dingqiang Zhu, Zitong Feng, Wenqiang Qi, Hui Tian, Cun Gao

**Affiliations:** 1 Department of Thoracic Surgery, Qilu Hospital of Shandong University, Jinan, Shandong China; 2 Department of Thoracic Surgery, The First Affiliated Hospital of Shandong First Medical University & Shandong Provincial Qianfoshan Hospital, Jinan, Shandong China; 3 Department of Urology, Qilu Hospital of Shandong University, Jinan, Shandong China

**Keywords:** Non-small-cell lung cancer, Tumour biomarkers

## Abstract

Lung adenocarcinoma (LUAD) remains a major global health issue characterized by high incidence and mortality rates. RecQ-like helicase 4 (RECQL4), a member of the DNA helicase family, plays a crucial role in DNA replication, DNA damage repair, and tumor progression. However, its involvement and specific molecular mechanisms in LUAD progression have not been elucidated. Through this investigation, we found that RECQL4 expression was aberrantly elevated in clinical LUAD tissues, and higher levels of RECQL4 expression were associated with poor prognosis and worse clinicopathological characteristics in LUAD patients. Gain-of-function and loss-of-function studies demonstrated that RECQL4 promoted the proliferation, migration, and invasion abilities of LUAD cells. Subsequent gene set enrichment analysis (GSEA) and Kyoto Encyclopedia of Genes and Genomes (KEGG) pathway enrichment analysis confirmed that RECQL4 activates the NF-κB signaling pathway. Mechanistic investigation indicated that RECQL4 might function as a scaffold protein for the Y box binding protein 1 (YBX1) and GTPase-activating protein SH3 domain-binding protein 1 (G3BP1), enhancing the interaction between YBX1 and G3BP1, thereby activating the NF-κB signaling pathway and promoting the progression of LUAD. In conclusion, RECQL4 promotes the malignant progression of LUAD through the YBX1/G3BP1-mediated NF-κB signaling pathway. These findings suggest that RECQL4 has the potential to serve as a novel prognostic biomarker and an effective therapeutic target for LUAD.

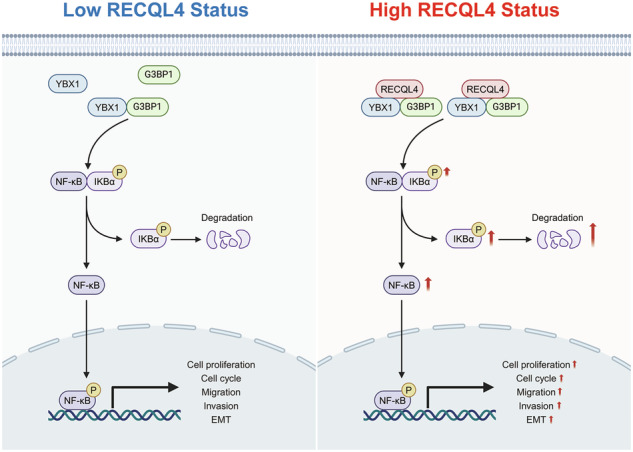

## Introduction

Non-small cell lung cancer (NSCLC) remains a major global health issue, marked by elevated incidence and mortality rates [1]. Among the different histological subtypes, lung adenocarcinoma (LUAD) is recognized as the most common type [2, 3]. Despite progress in multidisciplinary approaches to diagnosis and treatment that have led to some enhancement in clinical outcomes, the prognosis for patients with LUAD continues to be unfavorable [3]. In recent years, the rise of molecular targeted therapy and immunotherapy has significantly changed the diagnosis and treatment paradigm of lung cancer and substantially improved the prognosis of LUAD patients [4, 5]. Therefore, it is important to better understand the molecular mechanisms underlying LUAD initiation and progression and to identify novel prognostic biomarkers and effective therapeutic targets.

RecQ-like helicase 4 (RECQL4), a member of the RecQ helicase family characterized by its deoxyribonucleic acid (DNA)-unwinding capabilities, is crucial for preserving the stability of both nuclear and mitochondrial genomes [6, 7]. RECQL4 facilitates both nonhomologous end joining and homologous recombination processes [8]. Mutations and aberrant expression of RECQL4 can disrupt DNA repair mechanisms, resulting in the accumulation of DNA damage, an increase in genomic instability, and, consequently, the promotion of tumorigenesis [7, 9]. Accumulating evidence indicates that RECQL4 functions as an oncogene and facilitates the advancement of multiple cancer types. In gastric cancer, RECQL4 facilitated cisplatin resistance through the activation of an AKT–YB1–MDR1 signaling pathway [10]. In ovarian cancer, RECQL4 promoted the malignant progression and reduced chemotherapy sensitivity by increasing MAFB expression [11]. In cervical cancer and colon adenocarcinoma, the upregulation of RECQL4 enhanced cell malignant phenotypes via the PI3K/AKT signaling pathway [12, 13]. However, the prognostic and oncogenic significance of RECQL4 in LUAD has not been elucidated.

In this study, we found that RECQL4 expression was aberrantly elevated in both clinical LUAD tissues and tumor cell lines, and higher levels of RECQL4 expression were associated with poor prognosis and worse clinicopathological characteristics in LUAD patients. Cell functional experiments suggested that RECQL4 promoted the proliferation, migration, and invasion abilities of LUAD cells. In terms of mechanism, RECQL4 might function as a scaffold protein for the Y box binding protein 1 (YBX1)/ GTPase-activating protein SH3 domain-binding protein 1 (G3BP1) complex, enhancing the interaction between YBX1 and G3BP1, thereby activating the NF-κB pathway and promoting the progression of LUAD. Our findings suggest that RECQL4 has the potential to serve as a novel prognostic biomarker and an effective therapeutic target for LUAD.

## Results

### RECQL4 was significantly elevated and correlated with poor prognosis in LUAD patients

**Fig. 1:**
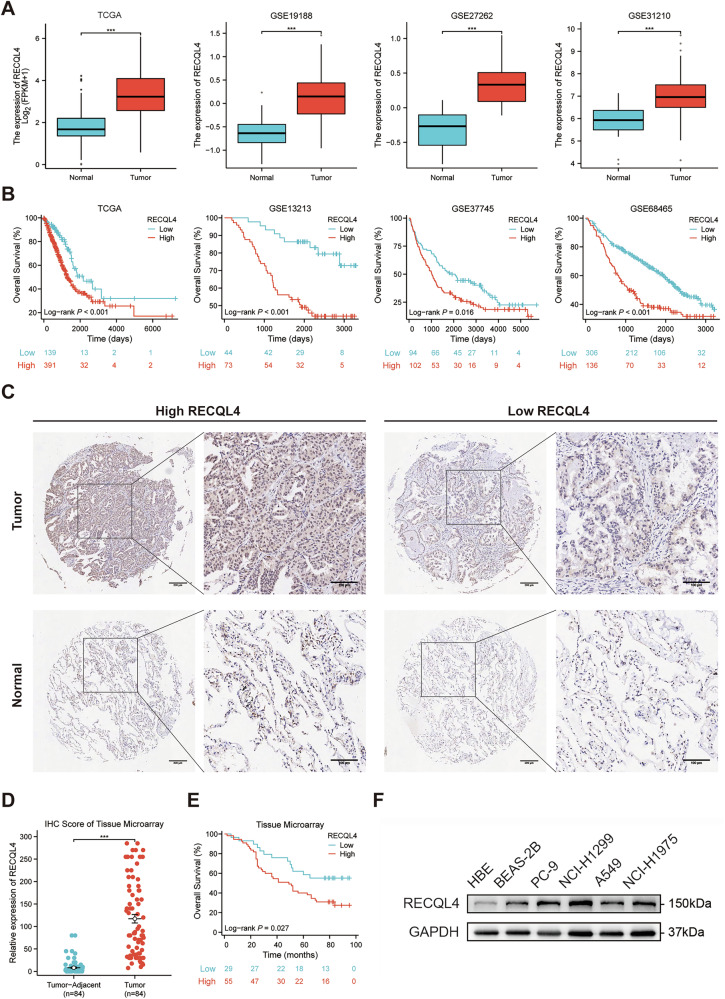
RECQL4 is significantly elevated in LUAD tissues and is associated with a poorer prognosis in LUAD patients. **A** The relative expression of RECQL4 in LUAD tissues compared to normal lung tissues according to TCGA and GEO databases. **B** Kaplan–Meier survival analysis based on RECQL4 expression using data from the TCGA and GEO databases. **C** Representative tissue microarray immunohistochemical images of RECQL4 expression in LUAD tissues and tumor-adjacent tissues. **D** IHC score of the RECQL4 expression in the tissue microarray. **E** Kaplan–Meier survival analysis based on RECQL4 expression using data from the tumor microarray. **F** Western blotting assays showing the expression of RECQL4 in 2 human bronchial epithelial cells and 4 LUAD cell lines. *p < 0.05, **p < 0.01, ***p < 0.001, ****p < 0.0001; RECQL4 RecQ like helicase 4, LUAD lung adenocarcinoma, TCGA The Cancer Genome Atlas, GEO Gene Expression Omnibus, IHC immunohistochemistry.

First, pan-cancer analysis indicated that RECQL4 was highly expressed in various types of cancers, including LUAD, esophageal squamous cell carcinoma and so on (Supplementary Fig. 1A). We then compared the mRNA level of RECQL4 in LUAD tissues and normal lung tissues in TCGA and several GEO (GSE19188, GSE27262, and GSE31210) datasets. The results showed that the mRNA level of RECQL4 was significantly increased in LUAD tissues compared to normal lung tissues in both TCGA and GEO datasets (Fig. 1A and Supplementary Fig. 1B). The Kaplan–Meier survival analysis indicated that patients exhibiting elevated expression levels of RECQL4 demonstrated worse overall survival in both TCGA and GEO (GSE13213, GSE37745, and GSE68465) datasets (Fig. 1B and Supplementary Fig. 1C, D). Subsequently, we conducted IHC on a tissue microarray that included 84 pairs of LUAD tissues and corresponding tumor-adjacent tissues in order to assess the level of RECQL4 protein expression (Fig. 1C). According to the IHC analysis, the RECQL4 expression was significantly increased in LUAD tissues compared to the corresponding tumor-adjacent tissues (Fig. 1D). Additionally, the Kaplan–Meier survival analysis based on the tissue microarray indicated that LUAD patients with increased expression of RECQL4 tend to have a worse overall survival (Fig. 1E). Furthermore, we analyzed the correlation between RECQL4 expression and clinicopathological characteristics based on tissue microarray, and the results showed that RECQL4 expression was significantly related to age and histopathological grade (Supplementary Table 1). The univariate and multivariate Cox regression analyses based on the tissue microarray indicated that RECQL4 level and pT stage were independent prognostic factors for LUAD patients (Table 1). Moreover, qRT-PCR and WB assays showed that the expression of RECQL4 in four LUAD cell lines (A549, NCI-H1299, PC-9, and NCI-H1975) was significantly higher than that in human bronchial epithelial cells (Fig. 1F and Supplementary Fig. 2A). In conclusion, the results indicate that RECQL4 levels are significantly increased in LUAD tissues and are associated with a poor prognosis in LUAD patients.

**Table 1:** Cox proportional hazard regression analysis of the factors associated with overall survival in lung adenocarcinoma patients.

Variables	Univariate analysis			Multivariate analysis		
	HR	95% CI	*p* value	HR	95% CI	*p* value
Age (>60 vs. ≤60)	1.450	0.838–2.507	0.184			
Sex (Male vs. Female)	1.073	0.622–1.850	0.801			
pT Stage (3–4 vs. 1–2)	3.662	1.613–8.312	0.002	2.997	1.077–8.345	0.036
pN Stage (1–3 vs. 0)	1.514	0.863–2.656	0.148			
pTNM Stage (III–IV vs. I–II)	2.243	1.168–4.305	0.015	1.475	0.658–3.308	0.346
Histopathological Grade (3 vs. 1–2)	1.670	0.914–3.050	0.095			
RECQL4 Expression (High vs. Low)	1.995	1.064–3.743	0.031	2.068	1.095–3.906	0.025

*pT* pathological tumor, *pN* pathological lymph node, *pTNM* pathological tumor-node-metastasis, *HR* hazard ratio, *CI* confidence interval.

### RECQL4 promoted the proliferation, migration, and invasion of LUAD cells in vitro

**Fig. 2:**
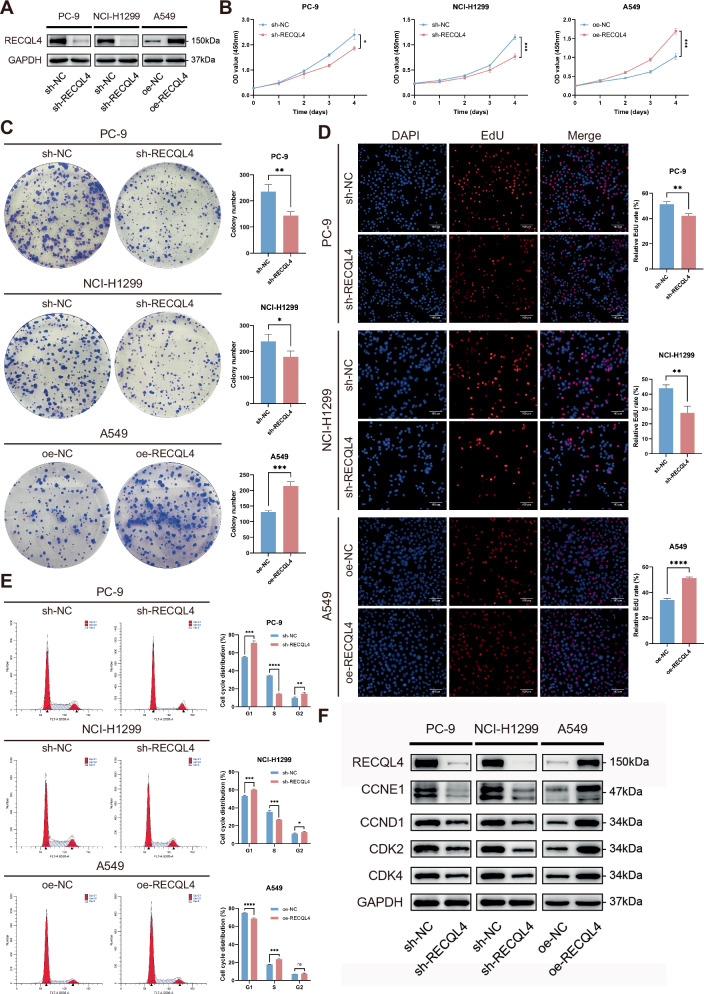
RECQL4 promotes the proliferation of LUAD cells in vitro. **A** The expression of RECQL4 was detected by western blotting after RECQL4 knockdown and overexpression. **B** CCK-8 assays, **C** Colony formation assays, **D** EdU incorporation assays, and **E** flow cytometry analyses were performed to evaluate the effects of RECQL4 on the proliferation ability of LUAD cells. **F** Western blotting assays showing the expression of cell-cycle-related proteins after RECQL4 knockdown or overexpression. *p < 0.05, **p < 0.01, ***p < 0.001, ****p < 0.0001; RECQL4 RecQ like helicase 4, LUAD lung adenocarcinoma, CCK-8 cell counting kit 8, EdU 5-ethynyl-2’-deoxyuridine.

**Fig. 3:**
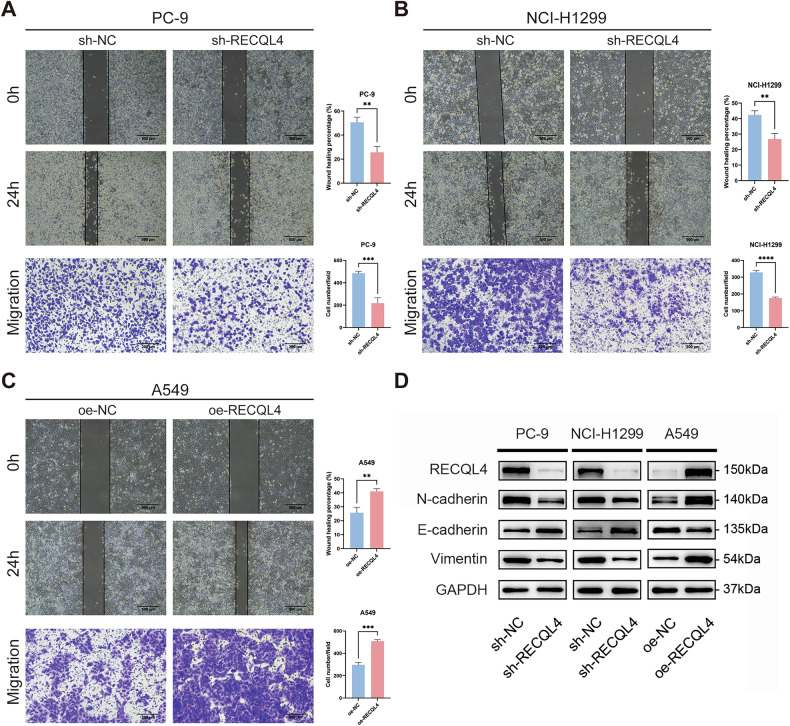
RECQL4 promotes the migration and invasion of LUAD cells in vitro. Wound-healing and Transwell assays were performed to evaluate the effects of RECQL4 on the migration and invasion abilities of **A** PC-9, **B** NCI-H1299, and **C** A549 LUAD cells. **D** Western blotting assays showing the expression of EMT regulatory proteins after RECQL4 knockdown or overexpression. *p < 0.05, **p < 0.01, ***p < 0.001, ****p < 0.0001; RECQL4 RecQ like helicase 4, LUAD lung adenocarcinoma, EMT epithelial-mesenchymal transition.

The results of WB and qRT-PCR assays showed that RECQL4 expression was higher in the PC-9 and NCI-H1299 cell lines, while the lowest expression was observed in the A549 cells among the four LUAD cell lines tested (Fig. 1F and Supplementary Fig. 2A). Consequently, we knocked down RECQL4 in the PC-9 and NCI-H1299 cells and overexpressed RECQL4 in the A549 cells. First, we transfected LUAD cells with two small interfering RNAs (siRNAs) targeting RECQL4 (si-RECQL4-1 and si-RECQL4-2) to knock down its expression. The results demonstrated that both mRNA levels and protein expression of RECQL4 were significantly reduced in PC-9 and NCI-H1299 cells (Supplementary Figs. 2B and 3A). Since si-RECQL4-1 most effectively suppressed RECQL4 expression, we used its sequence to generate a sh-RECQL4 lentivirus, thereby constructing stable RECQL4-knockdown LUAD cells. To examine the involvement of RECQL4 in LUAD progression, we developed the PC-9 and NCI-H1299 cell lines with stable knockdown of RECQL4 and the A549 cell line with stable overexpression of RECQL4 using lentiviruses to perform a range of cell functional experiments in vitro. We then assessed the efficacy of the knockdown and overexpression through qRT-PCR and WB assays. The results indicated a substantial decrease in RECQL4 expression in PC-9 and NCI-H1299 cell lines following stable knockdown, while a notable increase in RECQL4 expression was observed in A549 cells subsequent to stable overexpression (Fig. 2A and Supplementary Fig. 2C). Subsequently, CCK-8 assays, EdU assays, and colony formation assays were conducted to evaluate the effects of RECQL4 on cell proliferation. The results demonstrated a substantial reduction in the proliferation ability of the cells following the knockdown of RECQL4, whereas the overexpression of RECQL4 led to a marked enhancement in the proliferation ability of the cells (Fig. 2B–D and Supplementary Fig. 3B–D). Moreover, flow cytometry results indicated that the knockdown of RECQL4 resulted in a reduced percentage of cells in the S phase and an elevated percentage of cells in the G1 phase, while the overexpression of RECQL4 yielded the opposite effect (Fig. 2E and Supplementary Fig. 3E). WB assays were then conducted to validate significant alterations in cell-cycle-related proteins after RECQL4 knockdown or overexpression. We found that the expression of CDK2, CDK4, cyclin D1 (CCND1), and cyclin E1 (CCNE1) significantly decreased after RECQL4 knockdown in the PC-9 and NCI-H1299 cell lines, while the opposite results were observed after the overexpression of RECQL4 in the A549 cells (Fig. 2F and Supplementary Fig. 3F). Wound-healing assays and Transwell assays were conducted to investigate the effect of RECQL4 on the migration and invasion abilities of LUAD cells. The results showed that the knockdown of RECQL4 attenuated the migration and invasion abilities of LUAD cells, and the overexpression of RECQL4 resulted in the opposite effect (Fig. 3A–C and Supplementary Fig. 4A, B). Finally, we used WB assays to evaluate the alternation of some critical epithelial-mesenchymal transition (EMT) regulatory proteins. The results showed that the knockdown of RECQL4 led to the downregulation of N-cadherin and Vimentin, and the upregulation of E-cadherin, while the opposite results were observed after the overexpression of RECQL4 (Fig. 3D and Supplementary Fig. 4C). In conclusion, these above results indicate that RECQL4 functions as a tumor promoter, facilitating the proliferation, migration, and invasion of LUAD cells in vitro.

### RECQL4 facilitated the growth and metastasis of LUAD tumor in vivo

**Fig. 4:**
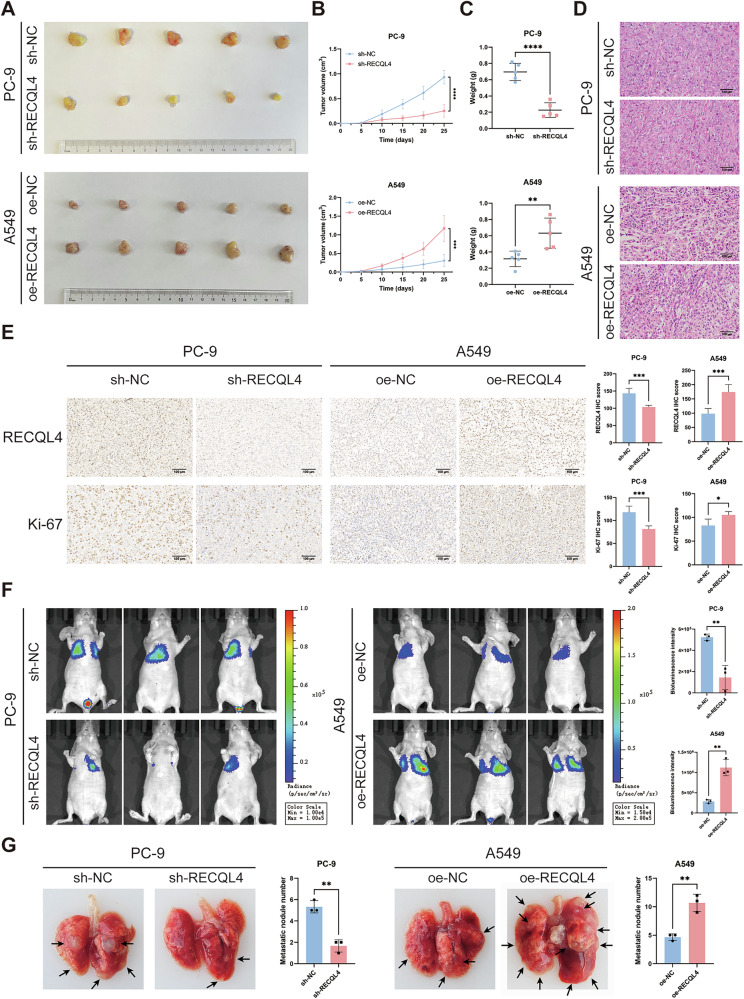
RECQL4 promotes the proliferation, migration, and invasion of LUAD cells in vivo. **A** Tumor pictures, **B** tumor growth curves, and **C** tumor weights in different groups (*n* = 5). **D** Representative images of H&E staining in different groups. **E** Representative images of immunohistochemical staining of RECQL4 and Ki-67 in different groups. **F** Lung metastatic lesions in different groups (*n* = 3) were evaluated by an in vivo bioluminescence imaging system. **G** Representative images of lung metastatic nodules in different groups. *p < 0.05, **p < 0.01, ***p < 0.001, ****p < 0.0001; RECQL4 RecQ like helicase 4, LUAD lung adenocarcinoma, H&E hematoxylin and eosin.

To further investigate the impact of RECQL4 on proliferation ability in vivo, we conducted xenograft subcutaneous tumor formation experiments utilizing BALB/c-nude mice. Nude mice were injected with PC-9 cells with stable knockdown of RECQL4 and the A549 cells with stable overexpression of RECQL4 and their corresponding negative control cells. The xenograft tumors exhibited a markedly reduced average size, growth rate, and weight in the sh-RECQL4 group in comparison to the sh-NC group. In contrast, the mice in the oe-RECQL4 group exhibited opposing results (Fig. 4A–C). Subsequently, we used H&E staining to assess tumor morphology, while IHC analysis was utilized to evaluate the expression levels of RECQL4 and Ki-67 (Fig. 4D, E). The results showed that the expression of RECQL4 and Ki-67 was significantly decreased in the sh-RECQL4 group, while the opposite result was observed in the oe-RECQL4 group. In addition, we developed a lung metastasis model using BALB/c-nude mice to further investigate the role of RECQL4 in lung metastasis in vivo. The results demonstrated that RECQL4 knockdown significantly reduced both lung bioluminescence intensity and the number of lung metastatic nodules. Conversely, RECQL4 overexpression markedly enhanced bioluminescent signals and increased pulmonary metastatic nodule formation (Fig. 4F, G). The above findings suggest that RECQL4 promotes the proliferation, migration, and invasion of LUAD cells in vivo.

### RECQL4 promoted LUAD progression by activating the NF-κB signaling pathway

**Fig. 5:**
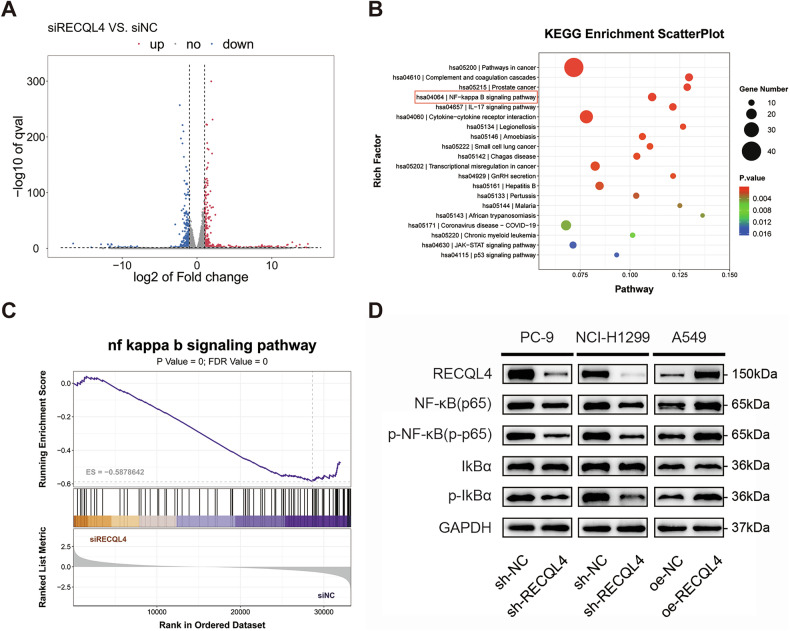
RECQL4 is closely associated with NF-κB signaling pathway in LUAD cells. **A** Volcano plot analysis of the transcriptome sequencing data. **B** KEGG pathway enrichment analysis of the transcriptome sequencing data. **C** GSEA enrichment analysis of NF-κB signaling pathway based on the transcriptome sequencing data. **D** Western blotting assays showing the expression of proteins associated with the NF-κB signaling pathway. *p < 0.05, **p < 0.01, ***p < 0.001, ****p < 0.0001; RECQL4 RecQ like helicase 4, LUAD lung adenocarcinoma, KEGG Kyoto Encyclopedia of Genes and Genomes, GSEA Gene Set Enrichment Analysis.

To elucidate the specific mechanism through which RECQL4 influences the progression of LUAD, we conducted transcriptome sequencing on PC-9 cells with RECQL4 knockdown, alongside a set of negative control cells. The results showed that the expression of 860 genes was significantly altered, including 429 genes that were downregulated and 431 genes that were upregulated (Fig. 5A). Additionally, the KEGG pathway and GSEA enrichment analysis indicated that NF-κB signaling pathway was significantly down-regulated after RECQL4 knockdown (Fig. 5B, C). Therefore, we assessed the alternation in the expression of NF-κB (p65), p-NF-κB (p-p65), IkBα, and p-IkBα after RECQL4 knockdown or overexpression using WB assays. The WB results showed that RECQL4 was positively related to the expression of NF-κB (p65), p-NF-κB (p-p65), and p-IkBα in the signaling pathway (Fig. 5D and Supplementary Fig. 4D). In addition, we performed IHC analysis to evaluate the expression levels of NF-κB (p65) in xenograft subcutaneous tumor, and the results showed that the expression of NF-κB (p65) was significantly decreased in the sh-RECQL4 group, while the opposite result was observed in the oe-RECQL4 group (Supplementary Fig. 5). These findings indicate that RECQL4 activates the NF-κB signaling pathway to promote LUAD progression.

### RECQL4 interacted with YBX1 and G3BP1

**Fig. 6:**
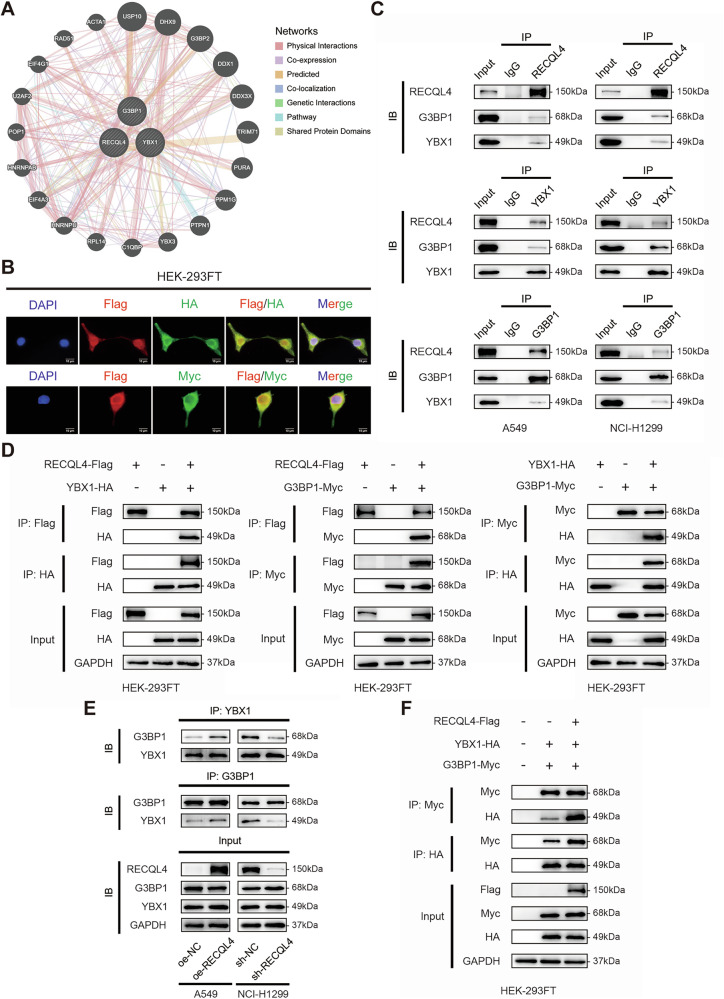
RECQL4 binds to the YBX1/G3BP1 complex and facilitates the interaction between YBX1 and G3BP1. **A** The protein–protein interaction network of RECQL4 was obtained from the GeneMANIA database. **B** Immunofluorescent staining was performed to confirm the colocalization of RECQL4, YBX1, and G3BP1. **C** Co-IP assays were performed in NCI-H1299 and A549 cells to examine the endogenous interaction among RECQL4, YBX1, and G3BP1. **D** HEK-293FT cells were co-transfected with RECQL4-Flag, YBX1-HA, and G3BP1-Myc, and Co-IP assays were performed to confirm the binding of the exogenous proteins. **E** Co-IP assays were performed to detect the binding affinity between endogenous YBX1 and G3BP1 after RECQL4 knockdown or overexpression. **F** Co-IP assays were performed to detect the binding affinity between exogenous YBX1-HA and G3BP1-Myc with or without RECQL4-Flag transfection. RECQL4 RecQ like helicase 4, YBX1 Y box binding protein 1, G3BP1 GTPase-activating protein SH3 domain-binding protein 1, Co-IP Co-immunoprecipitation.

We then focused on the proteins that may interact with RECQL4 to gain a deeper insight into the mechanism by which RECQL4 activates the NF-κB signaling pathway and further affects LUAD progression. First, we found that RECQL4 might interact with YBX1 and G3BP1 based on the GeneMANIA database (https://genemania.org/) (Fig. 6A). Moreover, a study has reported that the YBX1/G3BP1 complex could activate the downstream NF-κB signaling pathway in renal cell carcinoma cells [14]. Based on the results presented above, we identified YBX1 and G3BP1 as potential candidates to interact with RECQL4. First, we co-transfected RECQL4-Flag, YBX1-HA, and G3BP1-Myc into HEK-293FT cells and conducted IF to identify the colocalization of RECQL4 with YBX1 and G3BP1. We found that RECQL4 and YBX1/G3BP1 were predominantly colocalized in the cytoplasm (Fig. 6B). Subsequently, we performed MS and Co-IP to confirm whether RECQL4 interacted with YBX1 and G3BP1 (Fig. 6C, D and Supplementary Table 2). By Co-IP assays, we detected the formation of an immunocomplex among RECQL4, YBX1, and G3BP1 in endogenous proteins in both NCI-H1299 and A549 cells (Fig. 6C). To further confirm the result in exogenous proteins, we co-transfected RECQL4-Flag, YBX1-HA, and G3BP1-Myc into HEK-293FT cells and performed Co-IP assays using anti-Flag, anti-HA, and anti-Myc antibodies, and the same results were obtained (Fig. 6D). The results above demonstrate that RECQL4 interacts with and forms a complex with both YBX1 and G3BP1 in LUAD cells.

### RECQL4 bound to the YBX1/G3BP1 complex and enhanced their interaction

We first hypothesized that RECQL4 regulates the NF-κB signaling pathway by affecting YBX1 and G3BP1 expression levels, thereby promoting LUAD progression. However, the expression levels of YBX1 and G3BP1 were not altered by either knockdown or overexpression of RECQL4 (Fig. 6E, F and Supplementary Fig. 5). Since studies have proven that the binding of YBX1 and G3BP1 was essential in activation of the NF-κB signaling pathway [14, 15], we then hypothesized that RECQL4 may play a carcinogenic role by enhancing the interaction between YBX1 and G3BP1. Co-IP assays revealed that the knockdown of RECQL4 markedly decreased the YBX1-immunoprecipitated G3BP1, and G3BP1 similarly pulled down less YBX1 in RECQL4-knockdown cells in endogenous protein lysates, whereas RECQL4 overexpression significantly enhanced the interaction between YBX1 and G3BP1 (Fig. 6E). Subsequently, we co-transfected YBX1-HA and G3BP1-Myc into HEK-293FT cells with RECQL4 knockdown or overexpression for exogenous validation. Similarly, the results indicated that the combination of exogenous YBX1 and G3BP1 was elevated following the co-transfection of RECQL4-Flag (Fig. 6F). And the expression levels of endogenous or exogenous YBX1 and G3BP1 were almost unchanged, regardless of whether RECQL4 expression levels were increased or decreased (Fig. 6E, F). Altogether, these findings indicate that RECQL4 may serve as a scaffold protein for the YBX1/G3BP1 complex, facilitating the interaction between G3BP1 and YBX1, thereby activating the NF-κB pathway and promoting the progression of LUAD.

### Knockdown of YBX1 or G3BP1 partly rescued the tumor-promoting effects caused by RECQL4 overexpression in LUAD cells

**Fig. 7:**
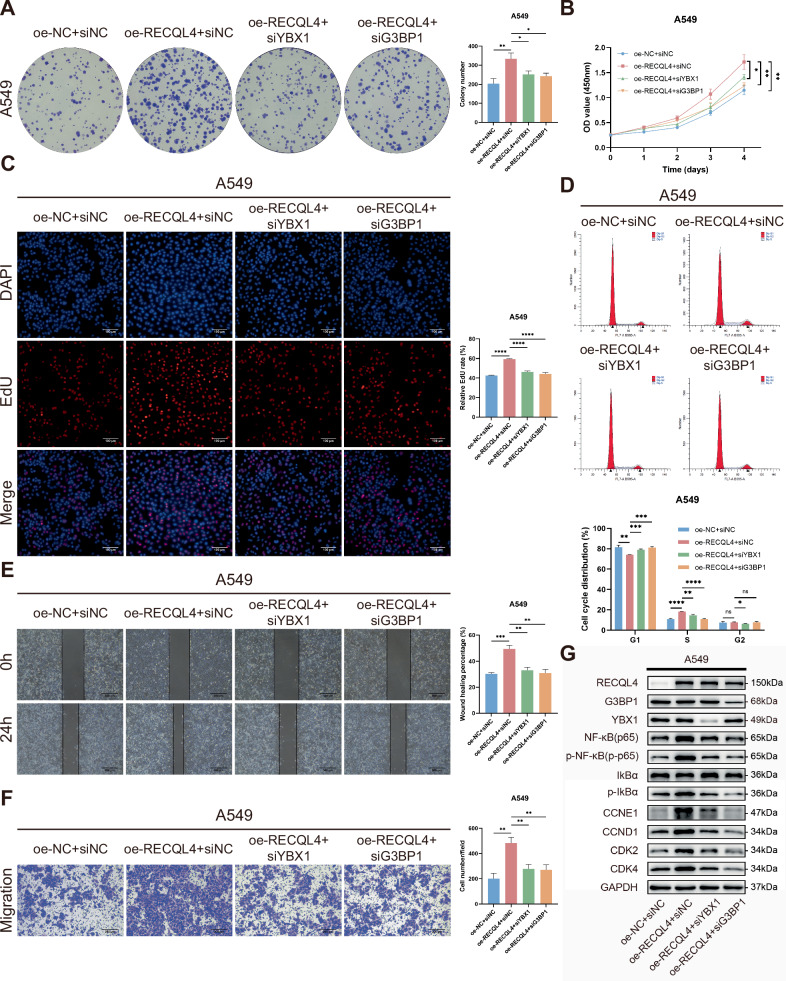
The knockdown of YBX1 or G3BP1 partially rescues the tumor-promoting effects caused by RECQL4 overexpression in LUAD cells. **A** Colony formation assays, **B** CCK-8 assays, **C** EdU incorporation assays, **D** flow cytometry analyses, **E** wound-healing assays, and **F** Transwell assays for the rescue experiments in different transfected groups. **G** Western blotting assays of NF-κB signaling pathways and cell-cycle-related markers were performed in the rescue experiments using A549 cells. *p < 0.05, **p < 0.01, ***p < 0.001, ****p < 0.0001; RECQL4 RecQ like helicase 4, YBX1 Y box binding protein 1, G3BP1 GTPase-activating protein SH3 domain-binding protein 1, LUAD lung adenocarcinoma, CCK-8 cell counting kit 8, EdU 5-ethynyl-2’-deoxyuridine.

We conducted additional experiments to investigate whether RECQL4 promotes the LUAD progression via YBX1 and G3BP1. The promoting effects of RECQL4 overexpression on A549 cell proliferation, cell cycle, migration, and invasion were partly rescued by the knockdown of either YBX1 or G3BP1 (Fig. 7A–F). As mentioned previously, the target proteins of the NF-κB signaling pathway and cell cycle were altered when RECQL4 was overexpressed. The WB assays showed that the inhibition of YBX1 or G3BP1 in A549 cells with RECQL4 overexpression reversed the alterations in the expression of target proteins related to the NF-κB signaling pathway and cell cycle caused by RECQL4 overexpression (Fig. 7G). In conclusion, RECQL4 promotes the malignant progression of LUAD through the YBX1/G3BP1-mediated NF-κB signaling pathway.

## Discussion

RECQL4 has been recognized as a DNA helicase that plays a vital role in preserving genomic stability and facilitating the repair of DNA damage [6]. In contrast to other human RecQ helicases that are exclusively localized within the nucleus, RECQL4 is present in both the nucleus and the cytoplasm [16, 17], indicating that RECQL4 might be involved in complicated regulatory networks beyond its role as a member of the RecQ helicase family. Recent studies have indicated that the overexpression of RECQL4 is common across various tumor types and is significantly associated with poorer clinical outcomes. However, the involvement and molecular mechanisms of RECQL4 in the LUAD progression remain unreported. In this study, we found that RECQL4 was significantly upregulated in patients with LUAD, and the overexpression of RECQL4 was associated with a poor prognosis. Furthermore, RECQL4 demonstrated significant oncogenic potential, enhancing the proliferation and metastatic capabilities of LUAD cells. Mechanistically, RECQL4 formed immunoprecipitants with YBX1/G3BP1 complex and enhanced the binding of YBX1 and G3BP1, thereby activating the NF-κB signaling pathway and promoting the LUAD progression. RECQL4 might serve as a crucial scaffold protein that contributes to the stability of the G3BP1/YBX1 complex. These findings suggest that RECQL4 has the potential to serve as a novel prognostic biomarker and an effective therapeutic target for LUAD.

Previous studies have reported that YBX1 and G3BP1 are associated with tumorigenesis, tumor progression, and resistance to chemotherapy in various types of cancer [18–21]. A study has reported that increased levels of G3BP1 are correlated with YBX1 and serve as a prognostic indicator of unfavorable outcomes in patients with NSCLC following surgical resection [22]. YBX1 is a well-conserved protein belonging to the RNA-binding protein family, and it performs a range of functions, including DNA repair, mRNA splicing, RNA stabilization, translation repression, and transcriptional regulation [23–25]. The YBX1 protein is composed of three independent domains: an amino-terminal region (N-terminal domain, aa 1–50), a cold shock domain (aa 51–129), and a carboxy-terminal region (C-terminal domain, aa 130–324) [26]. It has been proven that YBX1 is capable of directly interacting with G3BP1 via its C-terminal domain (aa 130-205) [14]. G3BP1 is an RNA-binding protein that plays a critical role in the regulation of various cellular functions [27]. Previous studies have demonstrated that G3BP1 is involved in the modulation of mRNA stability in response to external stimuli and is significant in the formation of stress granules [28–30]. However, the specific molecular biological mechanisms by which YBX1 and G3BP1 promote the development of LUAD have not yet been elucidated. In the present study, we found that RECQL4 promoted the progression of LUAD by activating the NF-κB signaling pathway through enhancing the interaction between YBX1 and G3BP1, rather than through altering their expression levels.

The NF-κB signaling pathway serves as a critical regulator of tumorigenesis and immune responses, facilitating cellular proliferation, enhancing cell migration and invasion, promoting angiogenesis and metastasis, and inhibiting apoptosis and cellular death [31]. Several studies have reported that YBX1 and G3BP1 facilitate the activation of the NF-κB signaling pathway through different mechanisms. For example, YBX1 promotes cell proliferation, invasion, and drug resistance through NF-κB signaling pathway in human neuroblastoma [32]. G3BP1 activates the NF-κB signaling pathway at the level of phosphorylation through cyclic GMP-AMP synthase (cGAS), thereby facilitating senescence-associated tumor growth [33]. In addition, the complex composed of YBX1 and G3BP1 plays a crucial role in enhancing NF-κB promoter activity and promoting the metastasis of renal cell carcinoma [14]. In this study, we first reported that RECQL4 promotes the malignant progression of LUAD through the YBX1/G3BP1-mediated NF-κB signaling pathway, providing novel insights into the mechanism of RECQL4-mediated LUAD progression.

Given the demonstrated roles of RECQL4 in maintaining genomic stability and facilitating cancer progression, targeting RECQL4 appears to be a promising strategy for cancer treatment [34]. However, this approach may adversely impact the survival and proliferation of normal healthy dividing cells in the body, leading to potential health risks. Therefore, any strategy that targets RECQL4 must be highly specific to cancer cells. Since most cells in the body, with the exception of adult stem cells, are primarily in a quiescent state, targeting the DNA replication machinery through RECQL4 may hold great promise for cancer treatment [7]. In fact, reducing the expression level of RECQL4 to that of normal cells is sufficient enough to abolish the tumorigenic potential of cancer cells [35]. Consequently, it is not necessary to entirely eliminate RECQL4 to achieve the destruction of cancer cells, which allows for the preservation of healthy normal cells. Considering the rapid proliferation of cancer cells, the development of novel tumor-specific targeting strategies aimed at DNA replication and repair factors, such as RECQL4, holds great promise for cancer treatment.

In conclusion, our study has identified DNA helicase RECQL4 as a novel oncogenic factor for LUAD. Increased expression levels of RECQL4 were found to enhance malignant cellular phenotypes via the YBX1/G3BP1-mediated NF-κB signaling pathway. These findings highlight the potential of RECQL4 to serve as a novel biomarker for the diagnosis of LUAD and as a promising target for therapeutic strategies.

## Materials and methods

### Clinical tissue specimen and tissue microarray

The tissue microarray of LUAD tissues (HLugA180Su11) was obtained from Shanghai Outdo Biotech (Shanghai, China), which contained 84 pairs of LUAD tissues and corresponding tumor-adjacent tissues. The Ethics Committee of Shanghai Outdo Biotech Company has approved the project (No. SHYJS-CP-2206001). Immunohistochemistry (IHC) staining was conducted, and the IHC scores were evaluated independently by two pathologists. X-tile software was employed to determine the optimal cut-off values for categorizing high and low expression groups.

### Cell culture and treatment

Two human bronchial epithelial cells (HBE and BEAS-2B), four LUAD cell lines (A549, NCI-H1299, PC-9, and NCI-H1975), and human embryonic kidney cells (HEK-293FT) were obtained from the Shanghai Cell Bank Research Center, Chinese Academy of Sciences (Shanghai, China). The cells were cultured in Dulbecco’s modified Eagle medium (DMEM; BasalMedia, Shanghai, China) containing 10% fetal bovine serum (FBS; Gibco, NY, USA) and were incubated in a humidified incubator maintained at 37 °C with an atmosphere of 5% carbon dioxide. Short tandem repeat (STR) analysis was employed to identify all cell lines, and the cultures underwent routine testing for mycoplasma contamination at 3-month intervals. The cells underwent transient transfection with small interfering RNA (siRNA) or plasmid utilizing the jetPRIME transfection reagent (Polyplus, NY, USA), following the guidelines provided by the manufacturer. Approximately 48 h post-transfection, the cells were harvested and lysed to evaluate the efficiency of the transfection process. The siRNAs and their corresponding negative controls were obtained from GenePharma Co., Ltd. (Shanghai, China) and Research Cloud Biology (Jinan, China). Overexpression plasmids were obtained from Research Cloud Biology (Jinan, China). Lentiviral vector-based short hairpin RNA (shRNA) for the stable knockdown of RECQL4 and lentivirus for stable RECQL4 overexpression were obtained from Jikai Company (Shanghai, China). The PC-9 [multiplicity of infection (MOI) = 20] and NCI-H1299 (MOI = 10) cells were transfected with the RECQL4 knockdown lentivirus, and the A549 cells were transfected with the RECQL4 overexpression lentivirus (MOI = 20). The puromycin (2 ug/mL) was used to select the stably transfected cells for 7 days. The siRNA and shRNA sequences are presented in Supplementary Table 3.

### Cell counting kit-8 (CCK-8) proliferation assay

The transfected cells were cultured in 96-well plates for durations of 24, 48, 72, and 96 h. Subsequently, the Cell Counting Kit-8 (CCK-8; #K1018, APExBIO, Wuhan, China) was added to the cell cultures. Following a 2-h incubation period in the dark, the absorbance was quantified at 450 nm utilizing a microplate reader. This experiment was performed three times independently.

### 5-ethynyl-2’-deoxyuridine (EdU) incorporation assay

The transfected cells were incubated overnight in 96-well plates. EdU staining was performed using an EdU staining kit (#C0075S, Beyotime, Shanghai, China) following the manufacturer’s protocol. Subsequently, images were acquired using an inverted fluorescence microscope (Olympus, Tokyo, Japan). This experiment was repeated three times independently.

### Colony formation assay

The transfected cells were seeded into six-well plates and cultured until colonies became discernible to the naked eye. Following this, the cells were fixed using 4% paraformaldehyde for a duration of 30 min, stained with crystal violet for 30 min, and subsequently washed three times with phosphate-buffered saline (PBS). Images were captured with a digital camera. The experiment was conducted three times independently.

### Wound-healing assay

The transfected cells were cultured in six-well plates with complete medium until the cells were nearly confluent. Subsequently, straight scratches were created using a 200-μL pipette tip. Following three washes with PBS, the cells were incubated in serum-free medium. The wound area was photographed with a microscope (Olympus, Tokyo, Japan) at 0 and 24 h, and measured using ImageJ software. This experiment was performed three times independently.

### Transwell assay

The experiments were conducted utilizing transwell chambers within 24-well plates (8 μm; Corning, NY, USA). The transfected cells were inoculated into the upper chamber, which contained a serum-free medium, while a medium supplemented with 20% FBS was added into the lower chamber. Following a 24-h incubation period at 37 °C, the cells were carefully detached from the upper surface of the membrane using a cotton swab. The migrated cells were subsequently fixed in 4% paraformaldehyde at room temperature for a duration of 30 min and then stained with crystal violet dye for an additional 30 min. After washing three times with PBS, photographs were taken with a light microscope (Olympus, Tokyo, Japan). This experiment was repeated three times independently.

### Quantitative real time polymerase chain reaction (qRT-PCR) assay

Total cellular ribonucleic acid (RNA) was extracted using the Rapid Cellular RNA Extraction Kit (#AC0205-B, SparkJade, Jinan, China) and subsequently reverse transcribed into complementary DNA (cDNA) with the aid of the Reverse Transcription Kit (#AG11706, Accurate Biology, Changsha, China). qRT-PCR was conducted on a LightCycler® 480 Instrument II detection system (Roche, Basel, Switzerland) employing the SYBR Green Premix Pro Taq HS qPCR kit (#AG11701, Accurate Biology, Changsha, China). The following primers were obtained from Biosune Company (Shanghai, China): RECQL4 (forward 5’-CCGCATCTTCCACGGCATC-3’, reverse 5’-CAGGTGCAGGTATTTTCTCCAG-3’) and GAPDH (forward 5’-GCACCGTCAAGGCTGAGAAC-3’, reverse 5’-TGGTGAAGACGCCAGTGGA-3’). The expression levels of the target genes were evaluated utilizing the 2^−ΔΔCT^ method. The experiment was conducted three times independently.

### Western blotting (WB) assay

The cells were lysed on ice in RIPA buffer (#P0038, Beyotime, Shanghai, China) containing 1% protease and phosphatase inhibitors. This lysis process was conducted for a duration of 30 min, during which the samples were vortexed every 10 min to ensure thorough lysis. The samples underwent centrifugation at 12,000 rpm and a temperature of 4 °C for a duration of 15 min. Subsequently, the supernatant was retrieved, and the protein concentration was determined using a bicinchoninic acid assay (BCA) kit (#P0012, Beyotime, Shanghai, China). Protein lysates were separated using 10% sodium dodecyl sulfate polyacrylamide gel electrophoresis (SDS-PAGE) gels and then electrically transported to polyvinylidene difluoride (PVDF) membranes. Following a 1-h incubation at room temperature with 5% skim milk powder for blocking, the membranes were subsequently incubated with the primary antibody at 4 °C overnight. After incubation with the appropriate secondary antibodies for 1 h at room temperature, the protein bands were visualized using an enhanced chemiluminescence (ECL) detection Kit (#P0018M, Beyotime, Shanghai, China) in accordance with the manufacturer’s instructions. The antibodies used in this experiment are presented in Supplementary Table 4. This experiment was performed three times independently.

### Co-immunoprecipitation (Co-IP) assay

The cells were lysed using IP buffer (#P0013, Beyotime, Shanghai, China) containing 1% protease and phosphatase inhibitors, and the cellular lysates were collected and centrifuged at 12,000 rpm for 15 min at 4 °C. The supernatant was then retrieved, and the protein concentration was determined using a BCA kit. A certain amount of the supernatant was separated as the input group. Each 500 µg of the lysate was incubated separately with 1 µg of the corresponding IP antibody for 8 h at 4 °C. Next, 50 µL of protein A/G magnetic beads (#HY-K0202, MedChemExpress, Shanghai, China) were added to the mixture, followed by an overnight incubation at 4 °C. After the samples were collected, WB assays were performed to detect the corresponding proteins. The antibodies used in this experiment are presented in Supplementary Table 4.

### Immunofluorescence (IF) assay

The transfected cells were seeded onto coverslips coated with poly-D-lysine in 6-well plates and subsequently incubated at 37 °C for a duration of 24 h. Subsequently, the cells were then fixed with 4% paraformaldehyde for 20 min, permeabilized with 0.3% Triton X-100 in PBS for 20 min, and blocked with 3% BSA in PBS for 1 h at room temperature. After incubating with the primary antibodies overnight at 4 °C, the cells were stained with CoraLite 488- and 594-conjugated secondary antibodies for 1 h at room temperature. The coverslips were then counter-stained with DAPI and photographed with a fluorescence microscope (Olympus, Tokyo, Japan). The antibodies used in this experiment are presented in Supplementary Table 4.

### Hematoxylin and eosin (H&E) and immunohistochemistry (IHC) staining

Tissues derived from patients diagnosed with LUAD and xenograft tumors from nude mice were fixed in 4% paraformaldehyde at room temperature, dehydrated through a graded ethanol series, embedded in paraffin, and sectioned into 4-μm slices. For H&E staining, each tissue section underwent deparaffinization, rehydration, and subsequent staining with H&E. For IHC staining, tissue sections underwent dewaxing, rehydration, antigen retrieval, peroxidase blocking, and non-specific binding site blocking. Sections were then incubated overnight with primary antibodies at 4 °C, followed by secondary antibodies for 1 h at room temperature. After DAB and hematoxylin staining, positive cells were counted using a microscopy. The IHC scoring system was established based on the histochemistry score, which was calculated using the following formula: IHC score = percentage of positive cells (0 − 100%) × staining intensities (0 = none; 1 = weak; 2 = moderate; and 3 = strong).

### Flow cytometry

The transfected cells were cultivated in six-well plates, and cell cycle analyses were conducted via flow cytometry following a 48-h incubation period. The Cell Cycle Staining Kit (#CCS012, Multi Sciences, Hangzhou, China) was employed to assess the cell cycle in accordance with the manufacturer’s guidelines. Data were processed using Modfit LT 5.0 software. This experiment was performed three times independently.

### In vivo experiments

Four-week-old female BALB/c-nude mice were obtained from Beijing Vital River Laboratory Animal Technology Co., Ltd. and were randomly assigned to cages. To promote a suitable living environment, each cage housed five mice. The mice were randomly assigned to groups using a random number table. In order to establish a subcutaneous xenograft tumor model, we injected stably transfected PC-9 and A549 cells (5 × 10^6^ cells per mouse) into the right axillary region of the mice and monitored tumor size every 5 days. Tumor volume was calculated using the following formula: Volume = (length × width^2^)/2. Following a duration of approximately 4 weeks, the mice were euthanized, and the tumors were meticulously harvested and documented using a digital camera. Subsequently, the tumors underwent H&E staining as well as IHC staining analysis. For the in vivo lung metastasis model, a total of 1 × 10^6^ luciferase-stably transfected cells were intravenously injected into nude mice via the tail vein. After 8 weeks, all mice were euthanized, and their lungs were harvested for metastatic nodule assessment. Prior to euthanasia, the mice were injected intraperitoneally with D-Luciferin (150 mg/kg, 100 μL) and imaged 10 min later using the IVIS spectrum system (PerkinElmer, Hopkinton, MA, USA) to evaluate lung metastasis. All animal experiments were approved by the Animal Research Ethics Committee of Qilu Hospital of Shandong University (No. DWLL-202500028), in accordance with the institutional guidelines for the care and use of animals.

### Transcriptomic sequencing, mass spectrometry (MS) for protein characterization, and bioinformatics analysis

Transcriptomic sequencing was conducted by LC-Bio Technology Co., Ltd. (Hangzhou, China). The mRNA transcripts differences between RECQL4 knockdown PC-9 cells and negative control PC-9 cells were compared. The Kyoto Encyclopedia of Genes and Genomes (KEGG) pathway and Gene Set Enrichment Analysis (GSEA) enrichment analyses were conducted utilizing the OmicStudio platform (https://www.omicstudio.cn/tool) [36]. Protein A/G magnetic beads, which were co-immunoprecipitated with RECQL4 antibody and cell lysates containing proteins, were collected for mass spectrometry analysis to identify the proteins that interact with RECQL4. Mass spectrometry was conducted by Jikai Company (Shanghai, China). The RNA sequencing (RNA-Seq) data from The Cancer Genome Atlas (TCGA) and the Genotype-Tissue Expression (GTEx) database were procured from the UCSC Xena platform (https://xenabrowser.net/datapages/). The gene expression profiling datasets (GSE19188, GSE27262, GSE31210, GSE13213, GSE37745, GSE68465) were obtained from the Gene Expression Omnibus (GEO) database (https://www.ncbi.nlm.nih.gov/gds). We used the Xiantao Academic Online Website (https://www.xiantaozi.com) for bioinformatics analysis based on the R language. The protein-protein interaction (PPI) network of RECQL4 was downloaded from the GeneMANIA website (https://genemania.org/) [37].

### Statistical analysis

All data were presented as the mean ± standard deviation (SD) based on three separate experiments. The Student’s *t* test was employed to examine differences between the two groups, and one-way analysis of variance (ANOVA) was utilized to assess differences among more than two groups. Categorical comparisons were conducted using either the Pearson chi-squared test or Fisher’s exact test. X-tile software was employed to determine the optimal cut-off values. Kaplan–Meier survival analysis was conducted to compare survival outcomes, and the log-rank test was employed to assess differences between the groups. Univariate and multivariate Cox proportional hazards models were constructed to identify independent prognostic factors. A two-sided *p* < 0.05 was considered statistically significant. All statistical analyses were performed using GraphPad Prism version 9.0 (GraphPad Software Inc., La Jolla, CA, USA), SPSS version 25.0 (IBM, Armonk, NY, USA), and R version 4.3.1 (R Development Core Team, Vienna, Austria).

## Supplementary information

Supplementary Figure 1

Supplementary Figure 2

Supplementary Figure 3

Supplementary Figure 4

Supplementary Figure 5

Supplementary Figure Legends

Supplementary Table 1

Supplementary Table 2

Supplementary Table 3

Supplementary Table 4

Original western blots

## Data Availability

All data analyzed within this study are included either in the manuscript or in the Supplementary Materials. The data that support the findings of this study are available on request from the corresponding author.
