# Supplementary figures and images for: RECQL4 promotes the malignant progression of lung adenocarcinoma through the YBX1/G3BP1-mediated NF-κB signaling pathway
Source: Cell Death Discov. 2026 Jan 9;12:8. doi: 10.1038/s41420-025-02849-3 (PMC12789086; doi:10.1038/s41420-025-02849-3)

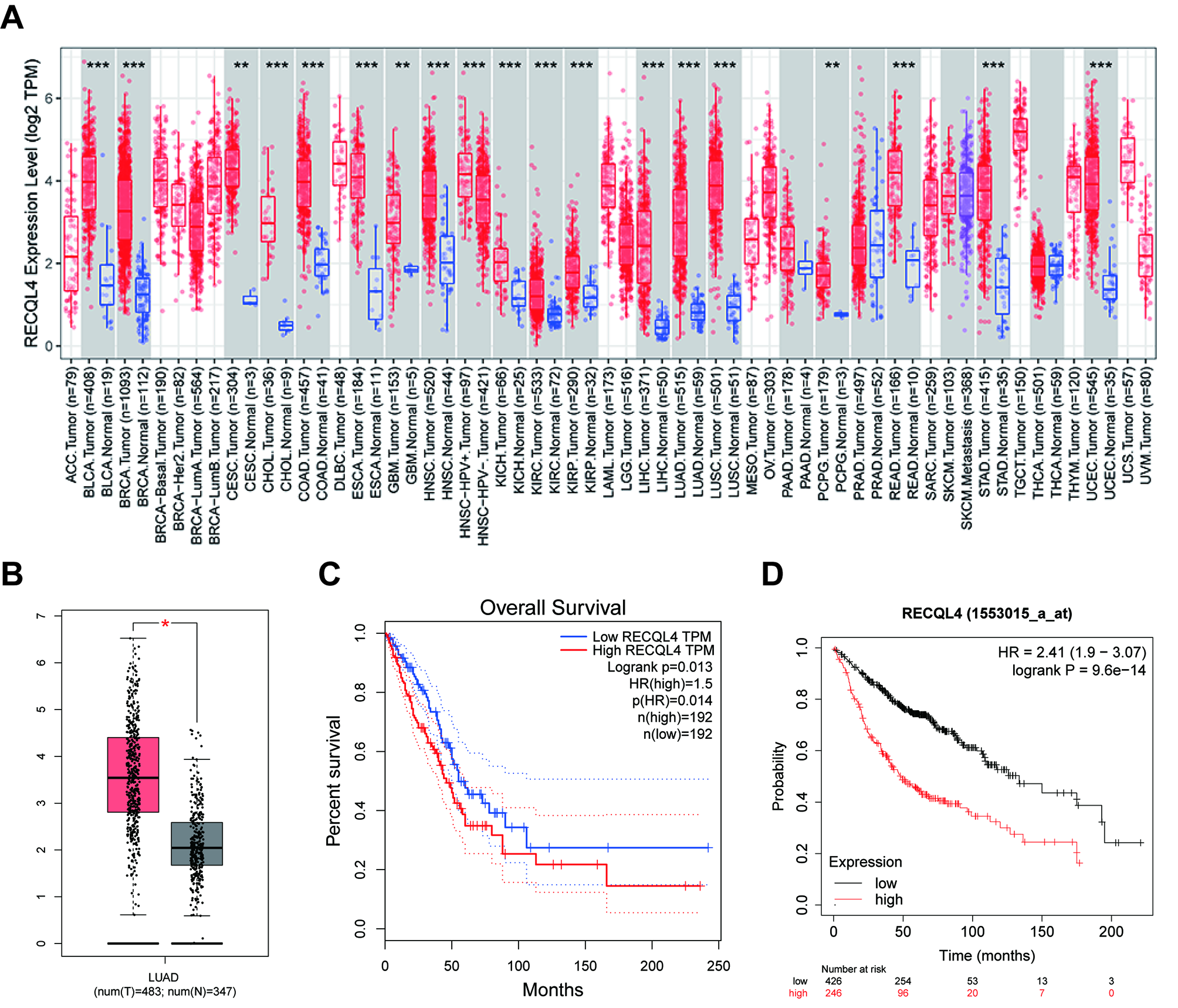

Supplement: Supplementary file 1 — Supplementary Figure 1 [file 41420_2025_2849_MOESM1_ESM.tif]

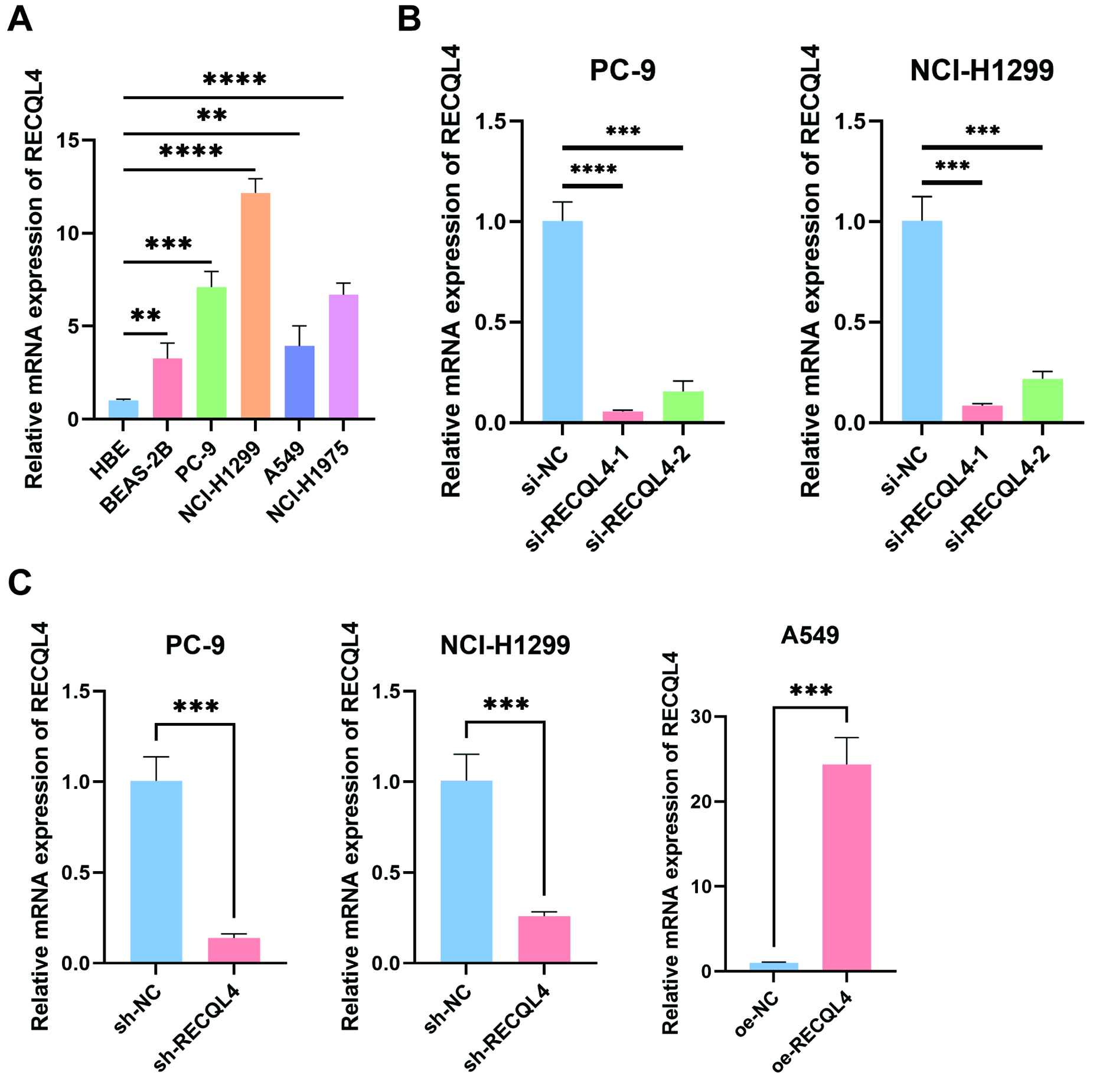

Supplement: Supplementary file 2 — Supplementary Figure 2 [file 41420_2025_2849_MOESM2_ESM.tif]

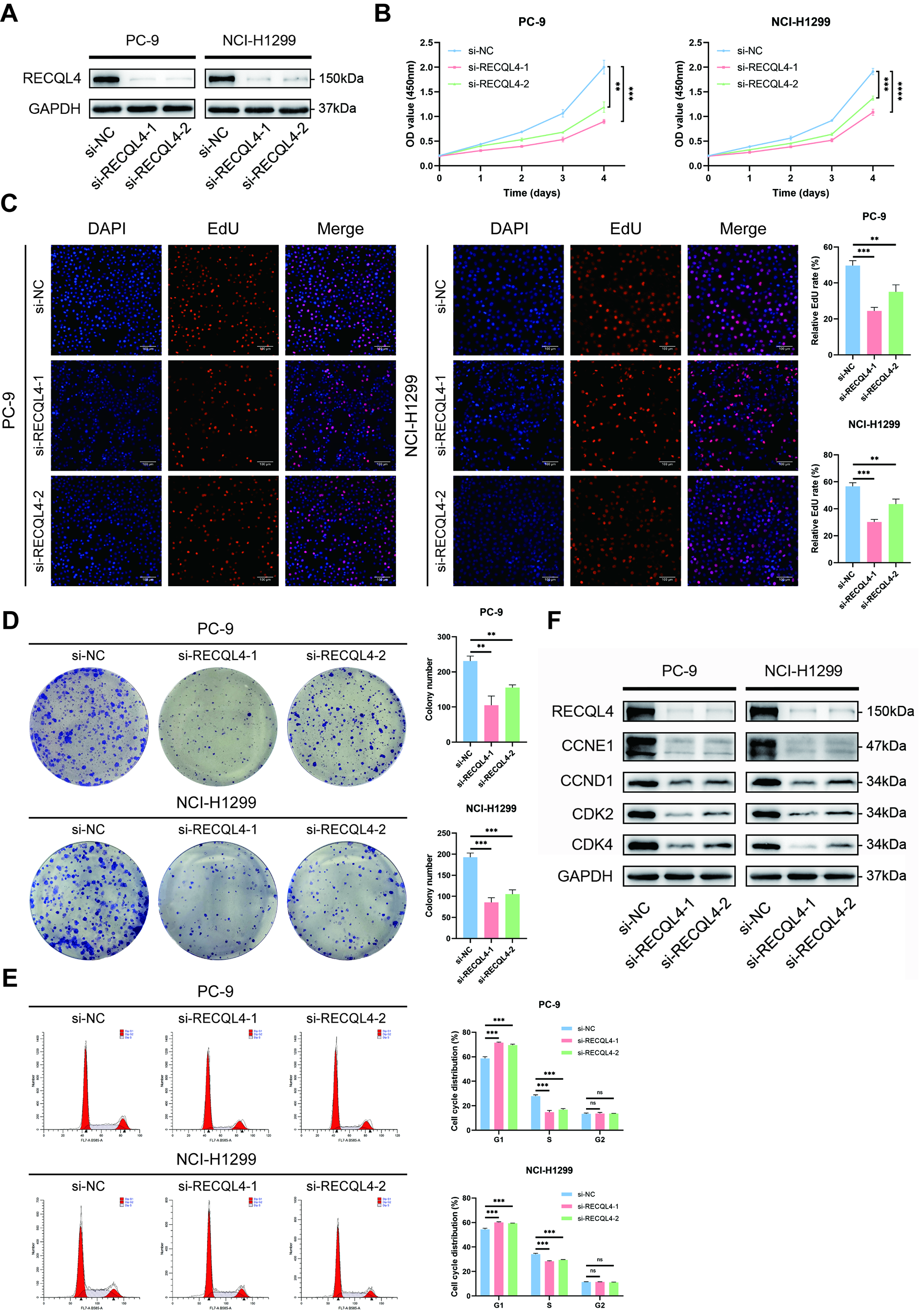

Supplement: Supplementary file 3 — Supplementary Figure 3 [file 41420_2025_2849_MOESM3_ESM.tif]

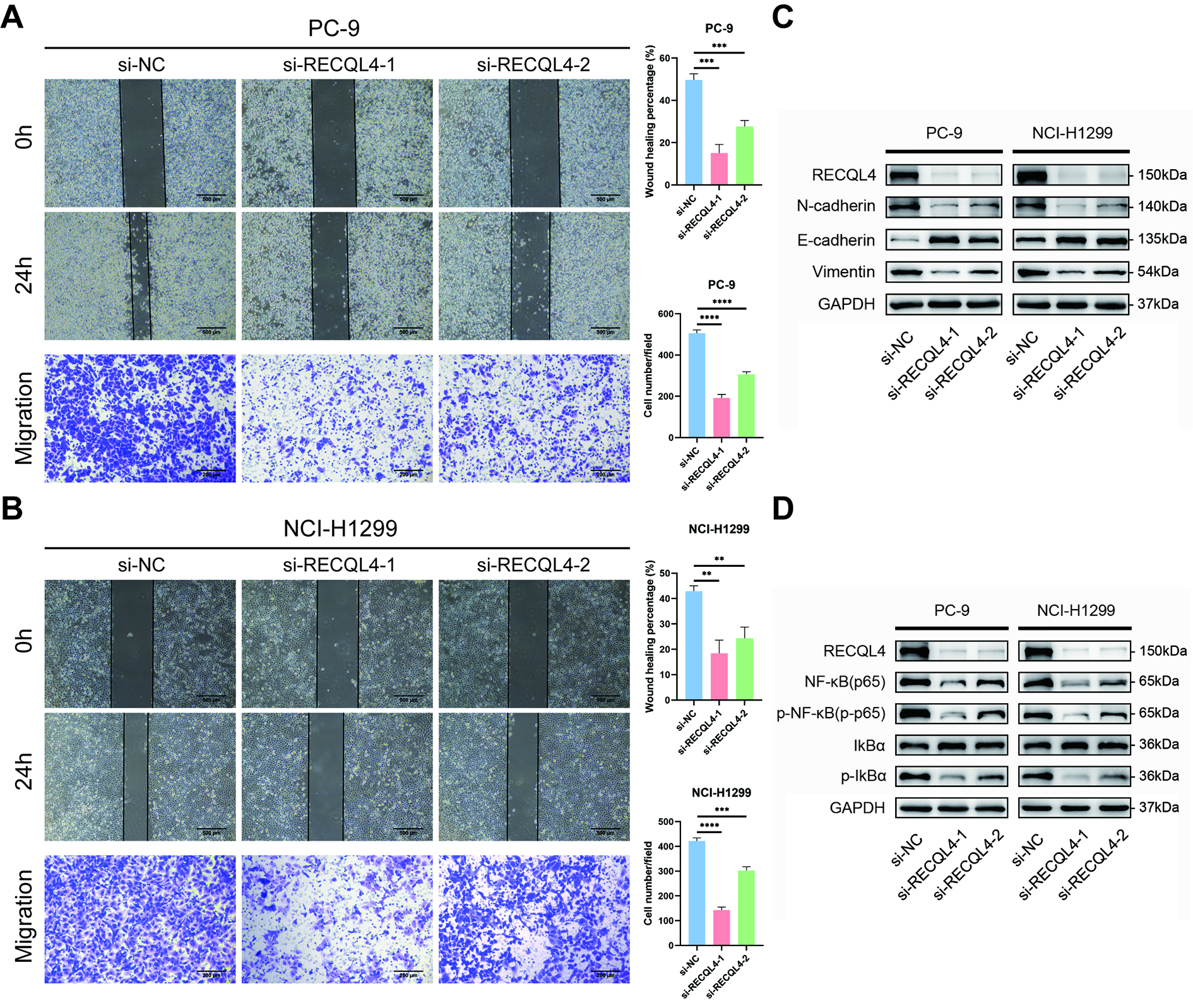

Supplement: Supplementary file 4 — Supplementary Figure 4 [file 41420_2025_2849_MOESM4_ESM.tif]

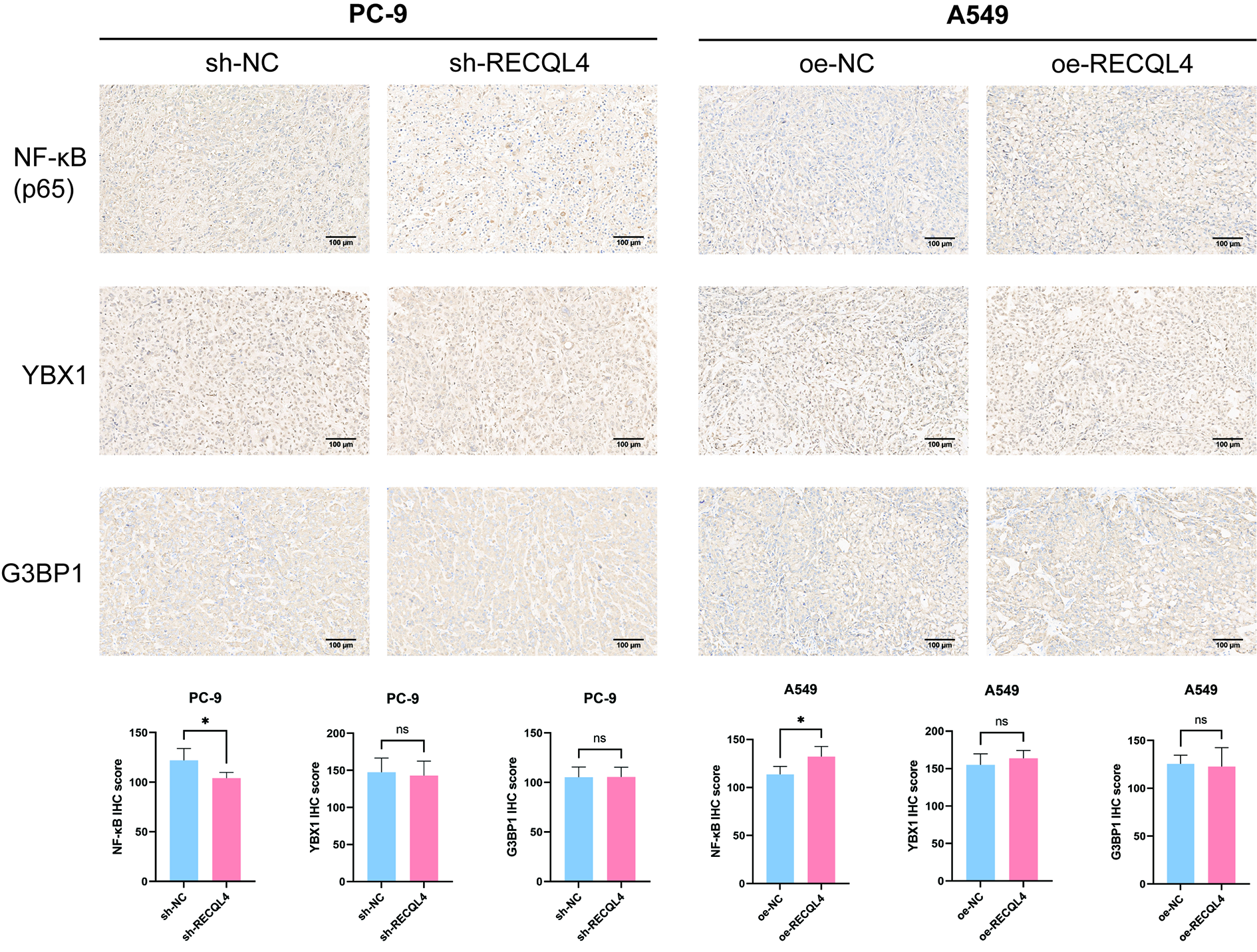

Supplement: Supplementary file 5 — Supplementary Figure 5 [file 41420_2025_2849_MOESM5_ESM.tif]
