# Supplementary material for: RECQL4 promotes the malignant progression of lung adenocarcinoma through the YBX1/G3BP1-mediated NF-κB signaling pathway
Source: Cell Death Discov. 2026 Jan 9;12:8. doi: 10.1038/s41420-025-02849-3 (PMC12789086; doi:10.1038/s41420-025-02849-3)
Supplement: Supplementary file 6 — Supplementary Figure Legends [file 41420_2025_2849_MOESM6_ESM.docx]

**Supplementary Figure 1.** The expression of RECQL4 is upregulated and is associated with poorer prognosis in LUAD patients. **(A)** The expression of RECQL4 was upregulated in a variety of cancers based on TIMER 2.0 database. **(B)** The relative expression of RECQL4 in LUAD tissues compared to normal lung tissues according to GEPIA database. **(C)** Kaplan-Meier survival analysis based on RECQL4 expression using data from the GEPIA database. **(D)** Kaplan-Meier survival analysis based on RECQL4 expression using data from the K-M plotter database. *P < 0.05, **P < 0.01, ***P < 0.001, ****P < 0.0001; RECQL4, RecQ like helicase 4; LUAD, lung adenocarcinoma.

**Supplementary Figure 2.** The knockdown and overexpression efficiency of RECQL4 is evaluated by qRT-PCR. **(A)** The expression of RECQL4 mRNA in 2 human bronchial epithelial cells and 4 LUAD cell lines. **(B)** The expression of RECQL4 was detected by qRT-PCR after RECQL4 knockdown by siRNAs. **(C)** The expression of RECQL4 was detected by qRT-PCR after RECQL4 stable knockdown and overexpression by lentivirus. *P < 0.05, **P < 0.01, ***P < 0.001, ****P < 0.0001; RECQL4, RecQ like helicase 4; LUAD, lung adenocarcinoma; qRT-PCR, quantitative real time polymerase chain reaction; siRNA, small interfering RNA.

**Supplementary Figure 3.** The knockdown of RECQL4 reduces the proliferation ability of LUAD cells in vitro. **(A)** The expression of RECQL4 was detected by western blotting after RECQL4 knockdown by siRNAs. **(B)** CCK-8 assays, **(C)** EdU incorporation assays, **(D)** Colony formation assays, and **(E)** flow cytometry analyses were performed to evaluate the proliferation ability of LUAD cells after RECQL4 knockdown by siRNAs. **(F)** Western blotting assays showing the expression of cell-cycle-related proteins after RECQL4 knockdown by siRNAs. *P < 0.05, **P < 0.01, ***P < 0.001, ****P < 0.0001; RECQL4, RecQ like helicase 4; LUAD, lung adenocarcinoma; CCK-8, cell counting kit 8; EdU, 5-ethynyl-2'-deoxyuridine; siRNA, small interfering RNA.

**Supplementary Figure 4.** The knockdown of RECQL4 reduces the migration and invasion abilities of LUAD cells in vitro. Wound-healing and Transwell assays were performed to evaluate the migration and invasion abilities of **(A)** PC-9, and **(B)** NCI-H1299 after RECQL4 knockdown by siRNAs. **(C)** Western blotting assays showing the expression of EMT regulatory proteins after RECQL4 knockdown by siRNAs. **(D)** Western blotting assays showing the expression of proteins associated with the NF-κB signaling pathway after RECQL4 knockdown by siRNAs. *P < 0.05, **P < 0.01, ***P < 0.001, ****P < 0.0001; RECQL4, RecQ like helicase 4; LUAD, lung adenocarcinoma; siRNA, small interfering RNA; EMT, epithelial-mesenchymal transition.

**Supplementary Figure 5.** Representative images of immunohistochemical staining for NF-κB (p65), YBX1, and G3BP1 in subcutaneous xenograft tumors across different groups. *P < 0.05, **P < 0.01, ***P < 0.001, ****P < 0.0001; RECQL4, RecQ like helicase 4; YBX1, Y box binding protein 1; G3BP1, GTPase-activating protein SH3 domain-binding protein 1.
