# Supplementary material for: RECQL4 promotes the malignant progression of lung adenocarcinoma through the YBX1/G3BP1-mediated NF-κB signaling pathway
Source: Cell Death Discov. 2026 Jan 9;12:8. doi: 10.1038/s41420-025-02849-3 (PMC12789086; doi:10.1038/s41420-025-02849-3)
Supplement: Supplementary file 7 — Supplementary Table 1 [file 41420_2025_2849_MOESM7_ESM.docx]

**Supplementary Table 1.** Correlation between RECQL4 expression and clinicopathological characteristics in patients diagnosed with lung adenocarcinoma.

| **Characteristics** | **RECQL4 Expression** | | ***P*** |
| --- | --- | --- | --- |
|  | **Low (n= 29)** | **High (n= 55)** |  |
| Age (years), n (%) |  |  | 0.003 |
| ≤60 | 21 (72.4) | 21 (38.2) |  |
| >60 | 8 (27.6) | 34 (61.8) |  |
| Sex, n (%) |  |  | 0.584 |
| Female | 15 (51.7) | 25 (45.5) |  |
| Male | 14 (48.3) | 30 (54.5) |  |
| Histopathological Grade, n (%) |  |  | 0.005 |
| Grade 1-2 | 27 (93.1) | 36 (65.5) |  |
| Grade 3 | 2 (6.9) | 19 (34.5) |  |
| pT Stage, n (%) |  |  | 0.838 |
| pT1-2 | 27 (93.1) | 49 (89.1) |  |
| pT3-4 | 2 (6.9) | 6 (10.9) |  |
| pN Stage, n (%) |  |  | 0.537 |
| pN0 | 17 (58.6) | 36 (65.5) |  |
| pN1-3 | 12 (41.4) | 19 (34.5) |  |
| pTNM Stage, n (%) |  |  | 0.373 |
| Stage I-II | 25 (86.2) | 43 (78.2) |  |
| Stage III-IV | 4 (13.8) | 12 (21.8) |  |

pT, pathological tumor; pN, pathological lymph node; pTNM, pathological tumor-node-metastasis.
