# Supplementary material for: RECQL4 promotes the malignant progression of lung adenocarcinoma through the YBX1/G3BP1-mediated NF-κB signaling pathway
Source: Cell Death Discov. 2026 Jan 9;12:8. doi: 10.1038/s41420-025-02849-3 (PMC12789086; doi:10.1038/s41420-025-02849-3)
Supplement: Supplementary file 9 — Supplementary Table 3 [file 41420_2025_2849_MOESM9_ESM.docx]

**Supplementary Table 3.** siRNA and shRNA used for cell transfection.

| **Name** | **Sense (5’-3’)** | **Antisense (3’-5’)** |
| --- | --- | --- |
| shNC | UUCUCCGAACGUGUCACGUTT | ACGUGACACGUUCGGAGAATT |
| shRECQL4 | GCACUCCCAAUACAGCUUATT | UAAGCUGUAUUGGGAGUGCTT |
| siNC | UUCUCCGAACGUGUCACGUTT | ACGUGACACGUUCGGAGAATT |
| siRECQL4-1 | GCACUCCCAAUACAGCUUATT | UAAGCUGUAUUGGGAGUGCTT |
| siRECQL4-2 | CCAUUAUCAUUUACUGCAATT | UUGCAGUAAAUGAUAAUGGTT |
| siYBX1 | GGAAUGACACCAAGGAAGATT | UCUUCCUUGGUGUCAUUCCTT |
| siG3BP1 | UCAAAGAGUGCGAGAACAATT | UUGUUCUCGCACUCUUUGATT |
