# Supplementary material for: RECQL4 promotes the malignant progression of lung adenocarcinoma through the YBX1/G3BP1-mediated NF-κB signaling pathway
Source: Cell Death Discov. 2026 Jan 9;12:8. doi: 10.1038/s41420-025-02849-3 (PMC12789086; doi:10.1038/s41420-025-02849-3)
Supplement: Supplementary file 10 — Supplementary Table 4 [file 41420_2025_2849_MOESM10_ESM.docx]

**Supplementary Table 4.** Antibodies used in the experimental assays.

| **Antigen** | **Supplier** | **Catalog** | **Application** |
| --- | --- | --- | --- |
| GAPDH | ABclonal | AC002 | WB (1:5000) |
| RECQL4 (Rabbit) | Proteintech | 17008-1-AP | WB (1:1000)  Co-IP (1μg/500μg lysate)  IHC (1:100) |
| RECQL4 (Mouse) | Santa Cruz | sc-518189 | WB (1:100)  Co-IP (1μg/500μg lysate) |
| YBX1 (Rabbit) | Proteintech | 20339-1-AP | WB (1:5000)  Co-IP (1μg/500μg lysate)  IHC (1:500) |
| YBX1 (Mouse) | Santa Cruz | sc-101198 | WB (1:200)  Co-IP (1μg/500μg lysate) |
| G3BP1 (Rabbit) | Proteintech | 13057-2-AP | WB (1:2000)  Co-IP (1μg/500μg lysate)  IHC (1:200) |
| G3BP1 (Mouse) | Santa Cruz | sc-365338 | WB (1:100)  Co-IP (1μg/500μg lysate) |
| CCNE1 | Huabio | ET1612-16 | WB (1:1000) |
| CCND1 | Huabio | ET1601-31 | WB (1:1000) |
| CDK2 | Proteintech | 10122-1-AP | WB (1:5000) |
| CDK4 | Huabio | ET1612-23 | WB (1:2000) |
| N-cadherin | Proteintech | 22018-1-AP | WB (1:2000) |
| E-cadherin | ABclonal | A20798 | WB (1:1000) |
| Vimentin | Huabio | R1308-6 | WB (1:1000) |
| NF-κB (p65) | Huabio | ET1603-12 | WB (1:1000)  IHC (1:100) |
| p-NF-κB (p65) | Abways | CY6372 | WB (1:1000) |
| IkBα | Proteintech | 10268-1-AP | WB (1:5000) |
| p-IkBα | Proteintech | 82349-1-RR | WB (1:1000) |
| Ki-67 | Servicebio | GB111499-100 | IHC (1:500) |
| Flag-Tag (Rabbit) | Huabio | 0912-1 | WB (1:5000)  Co-IP (1μg/500μg lysate)  IF (1:100) |
| Flag-Tag (Mouse) | Proteintech | 66008-4-Ig | WB (1:5000)  Co-IP (1μg/500μg lysate)  IF (1:100) |
| HA-Tag (Rabbit) | Proteintech | 51064-2-AP | WB (1:5000)  Co-IP (1μg/500μg lysate)  IF (1:100) |
| HA-Tag (Mouse) | ABclonal | AE008 | WB (1:2000)  Co-IP (1μg/500μg lysate)  IF (1:100) |
| Myc-Tag (Rabbit) | Huabio | R1208-1 | WB (1:1000)  Co-IP (1μg/500μg lysate)  IF (1:100) |
| Myc-Tag (Mouse) | Proteintech | 60003-2-Ig | WB (1:2000)  Co-IP (1μg/500μg lysate)  IF (1:100) |
| IgG | Abcam | ab6715 | Co-IP (1μg/500μg lysate) |
| HRP-anti-rabbit | Huabio | HA1001 | IB (1:50000) |
| HRP-anti-mouse | Huabio | HA1006 | IB (1:20000) |
| CoraLite488-conjugated Goat Anti-Rabbit IgG | Proteintech | SA00013-2 | IF (1:100) |
| CoraLite488-conjugated Goat Anti-Mouse IgG | Proteintech | SA00013-1 | IF (1:100) |
| CoraLite594-conjugated Goat Anti-Rabbit IgG | Proteintech | SA00013-4 | IF (1:100) |
| CoraLite594-conjugated Goat Anti-Mouse IgG | Proteintech | SA00013-3 | IF (1:100) |
