# Supplementary material for: RECQL4 promotes the malignant progression of lung adenocarcinoma through the YBX1/G3BP1-mediated NF-κB signaling pathway
Source: Cell Death Discov. 2026 Jan 9;12:8. doi: 10.1038/s41420-025-02849-3 (PMC12789086; doi:10.1038/s41420-025-02849-3)
Supplement: Supplementary file 11 — Original western blots [file 41420_2025_2849_MOESM11_ESM.docx]

**Figure 1F**

RECQL4 GAPDH


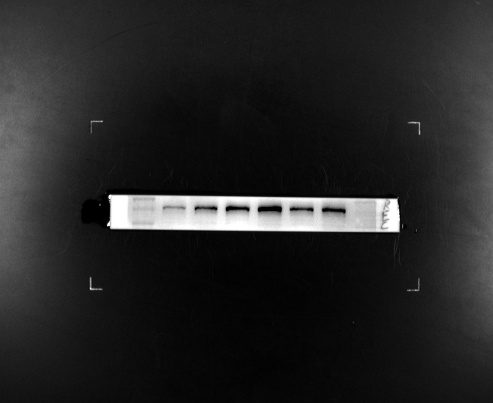

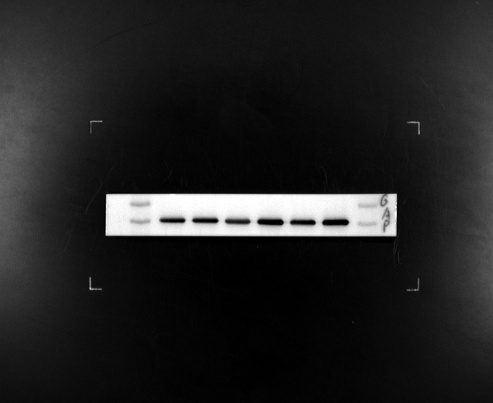


**Figure 2A**

RECQL4 (PC-9) GAPDH (PC-9)


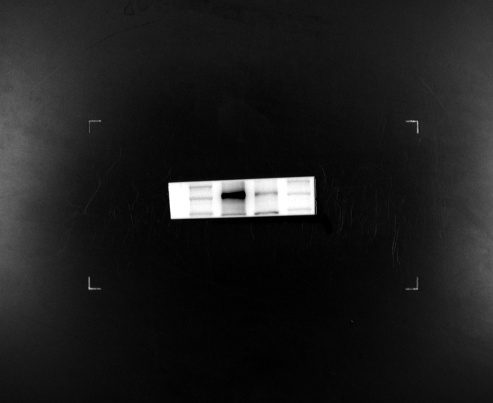

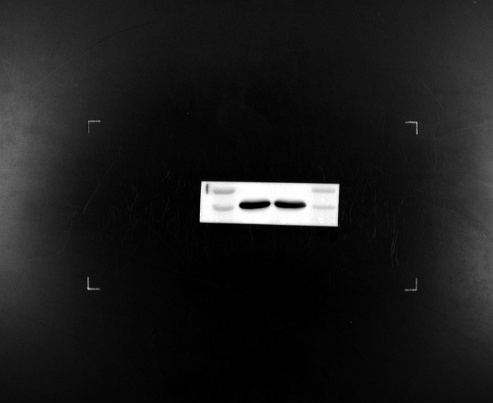


RECQL4 (NCI-H1299) GAPDH (NCI-H1299)


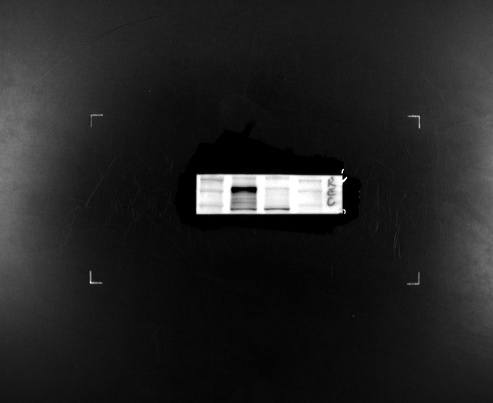

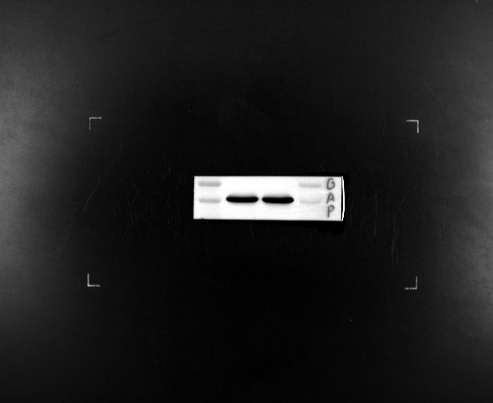


RECQL4 (A549) GAPDH (A549)


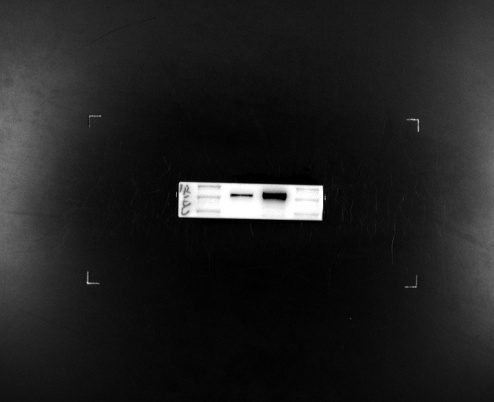

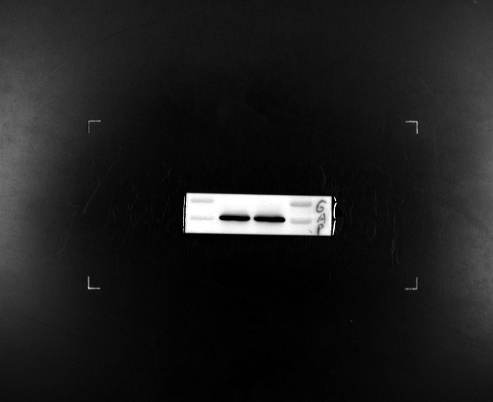


**Figure 2F**

RECQL4 (PC-9) CCNE1 (PC-9)


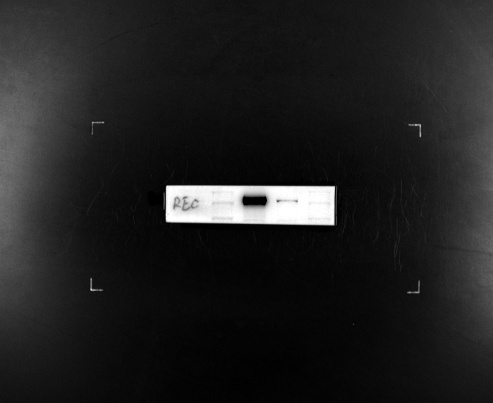

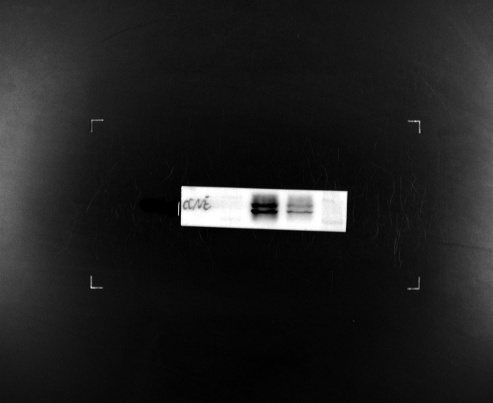


CCND1 (PC-9) CDK2 (PC-9)


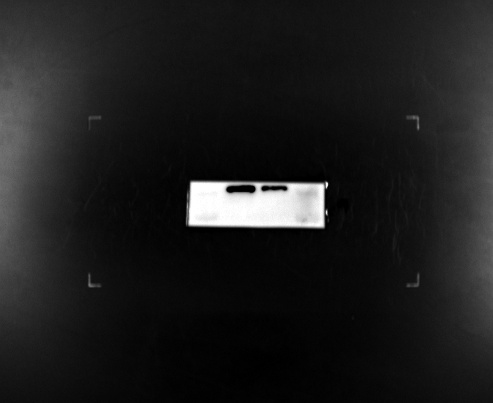

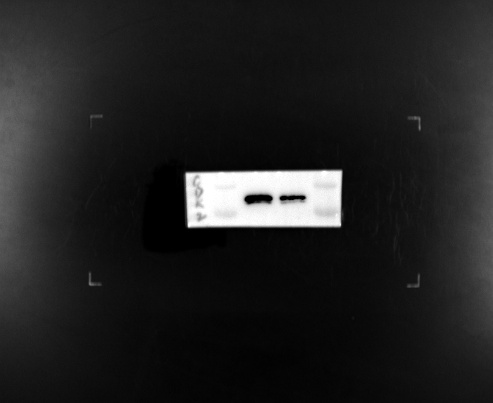


CDK4 (PC-9) GAPDH (PC-9)


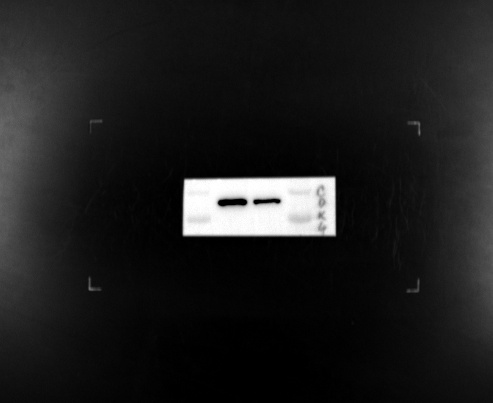

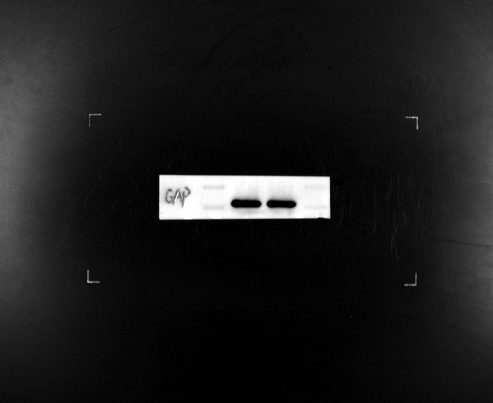


RECQL4 (NCI-H1299) CCNE1 (NCI-H1299)


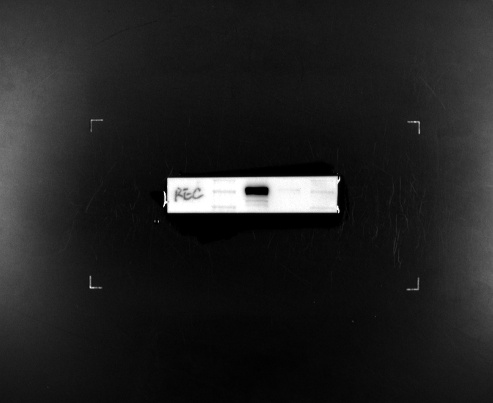

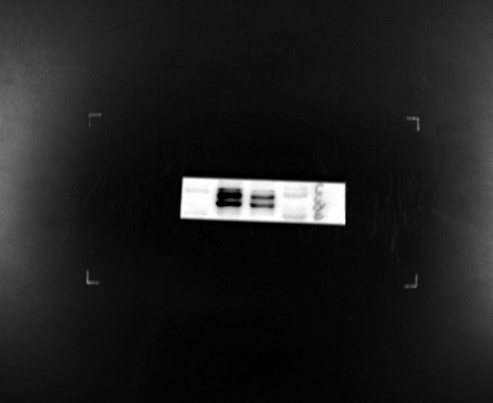


CCND1 (NCI-H1299) CDK2 (NCI-H1299)


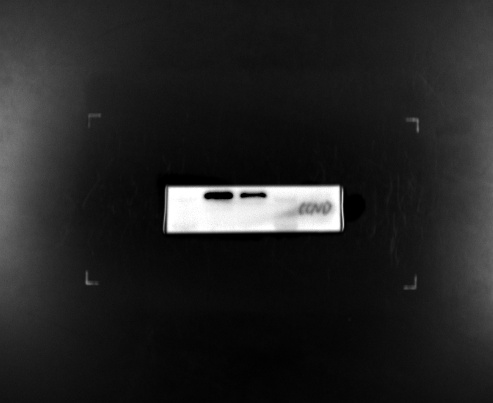

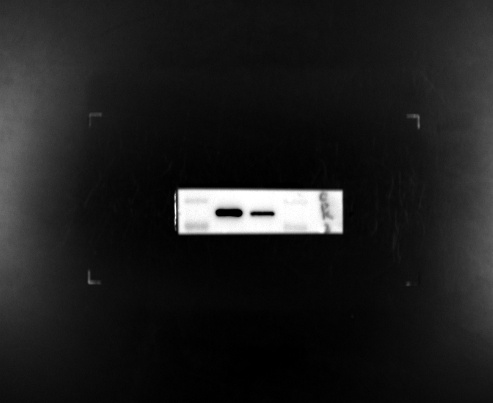


CDK4 (NCI-H1299) GAPDH (NCI-H1299)


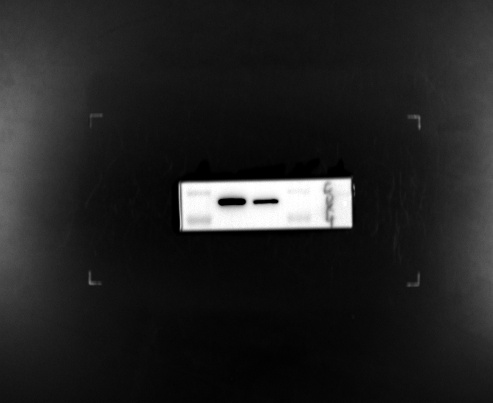

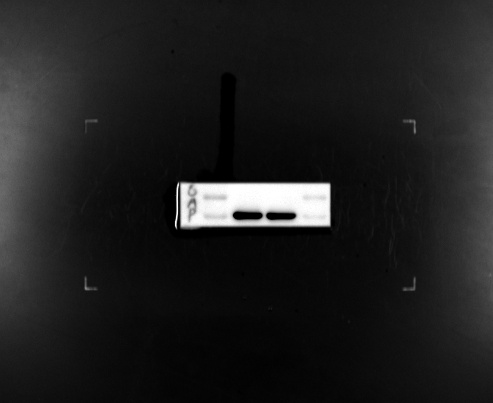


RECQL4 (A549) CCNE1 (A549)


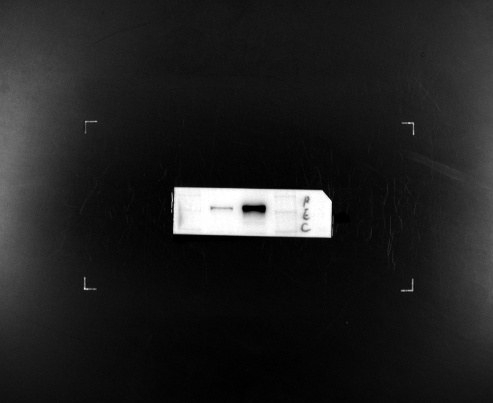

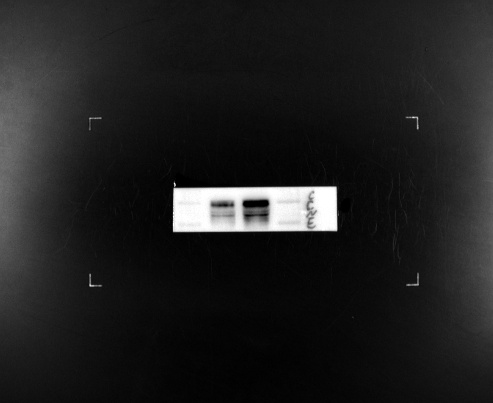


CCND1 (A549) CDK2 (A549)


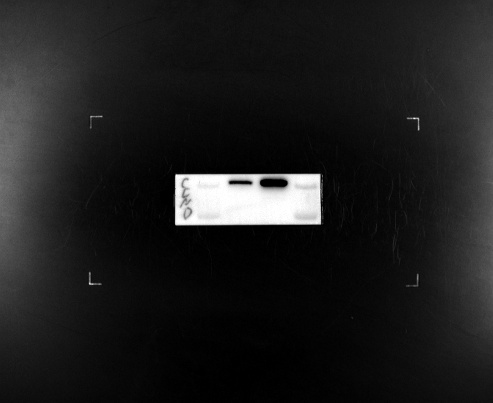

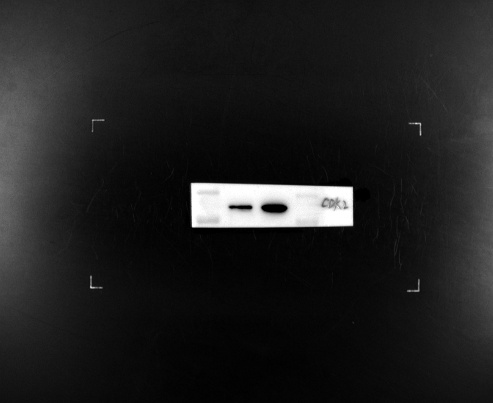


CDK4 (A549) GAPDH (A549)


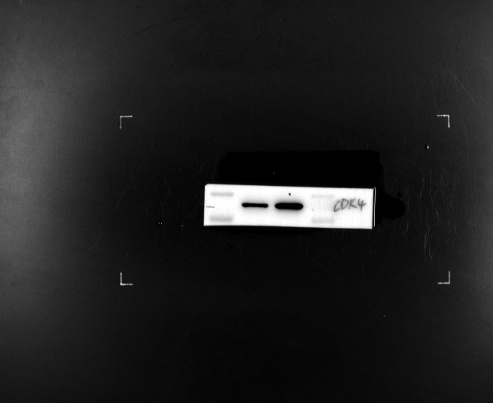

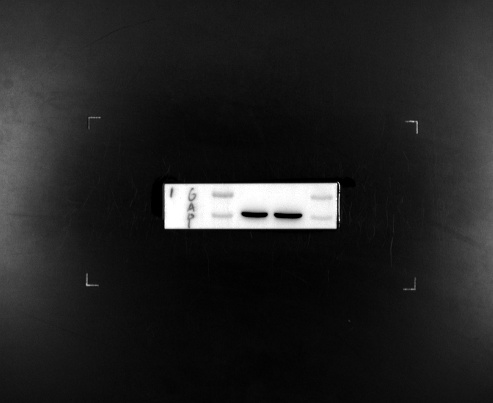


**Figure 3D**

RECQL4 (PC-9) N-cadherin (PC-9)


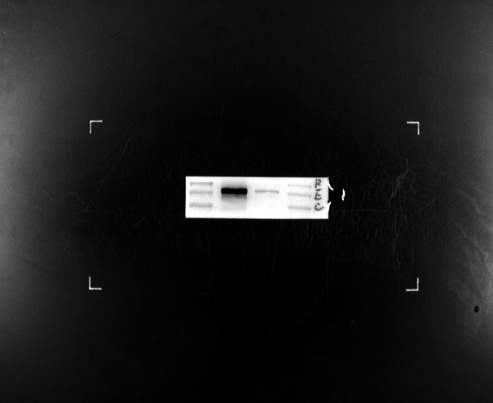

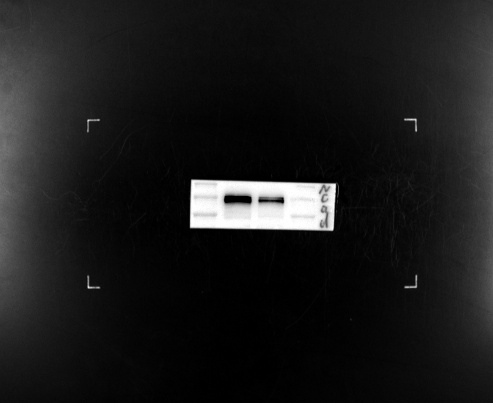


E-cadherin (PC-9) Vimentin (PC-9)


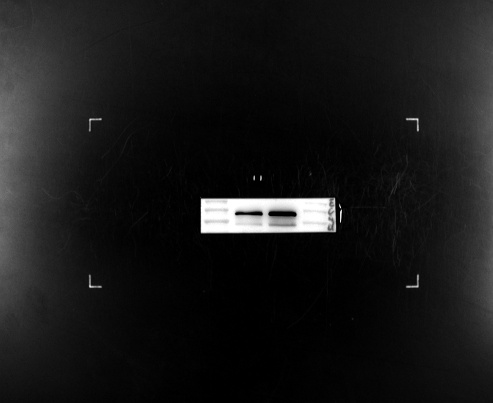

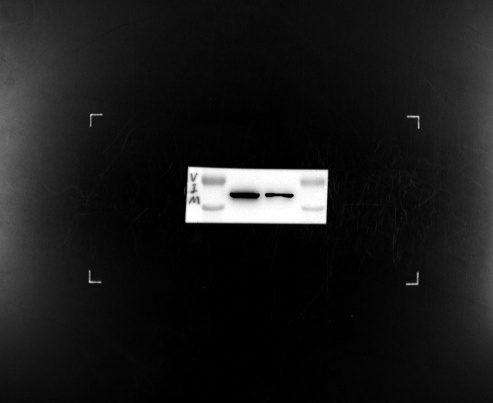


GAPDH (PC-9)


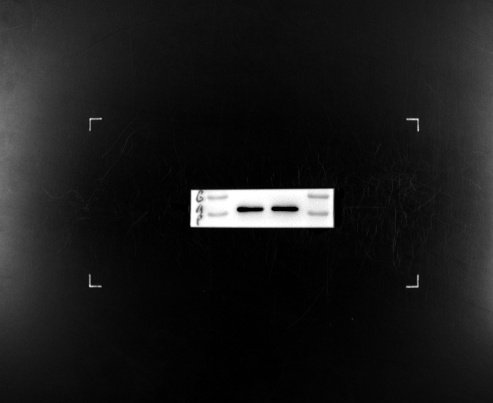


RECQL4 (NCI-H1299) N-cadherin (NCI-H1299)


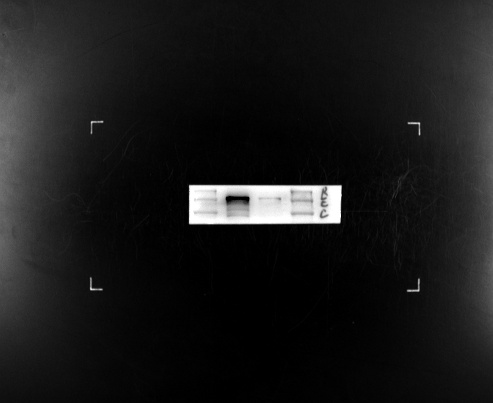

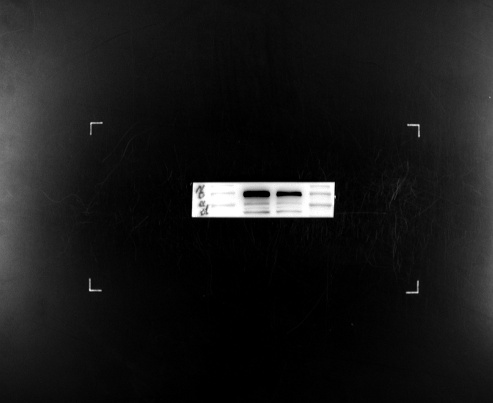


E-cadherin (NCI-H1299) Vimentin (NCI-H1299)


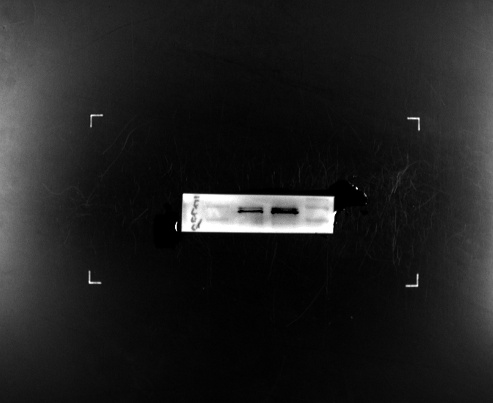

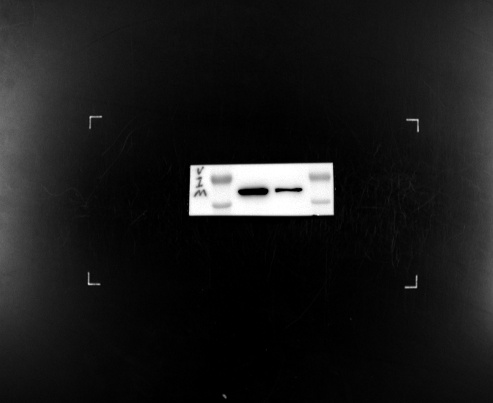


GAPDH (NCI-H1299)


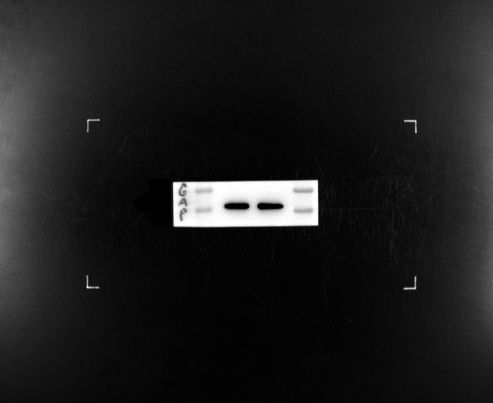


RECQL4 (A549) N-cadherin (A549)


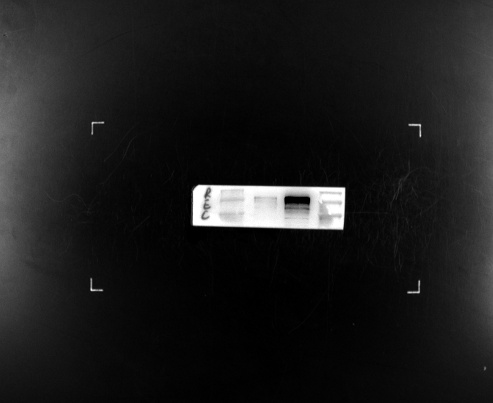

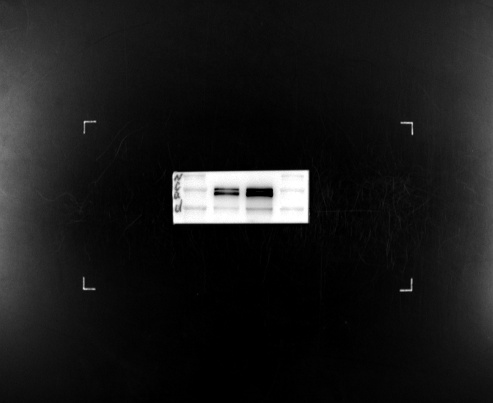


E-cadherin (A549) Vimentin (A549)


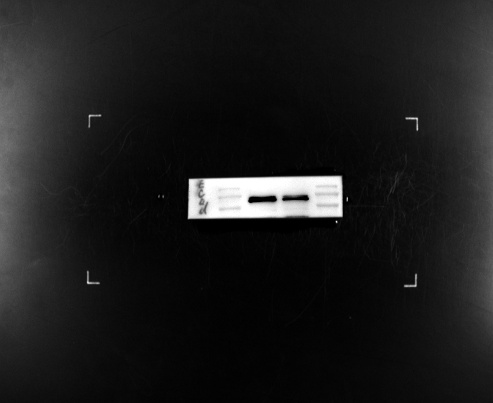

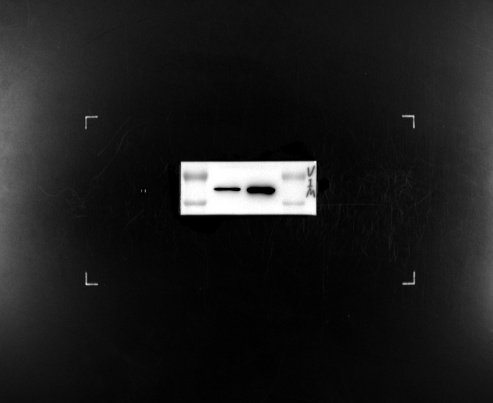


GAPDH (A549)


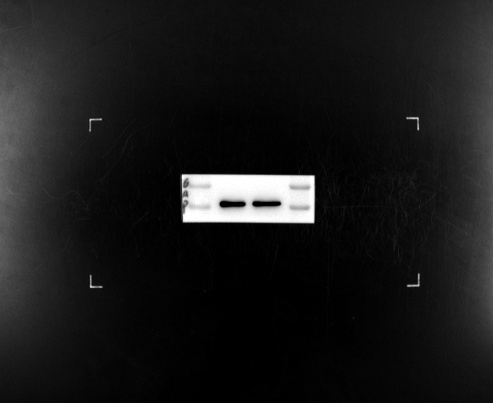


**Figure 5D**

RECQL4 (PC-9) NFκB (PC-9)


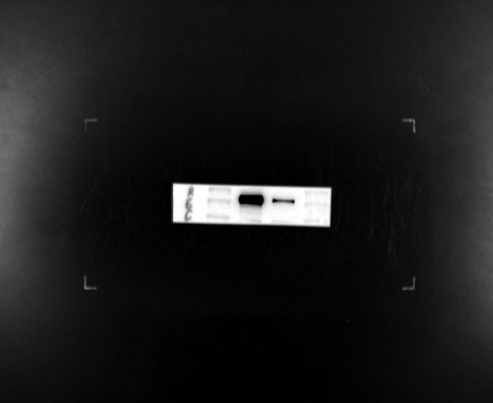

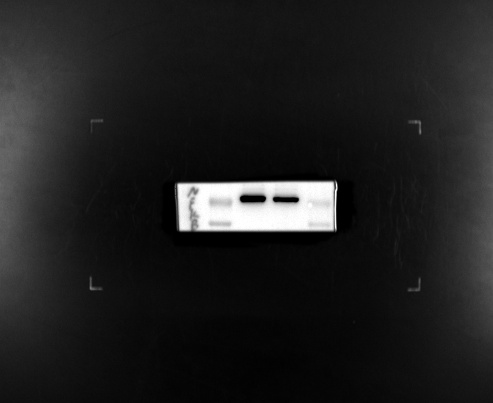


p-NFκB (PC-9) IkBα (PC-9)


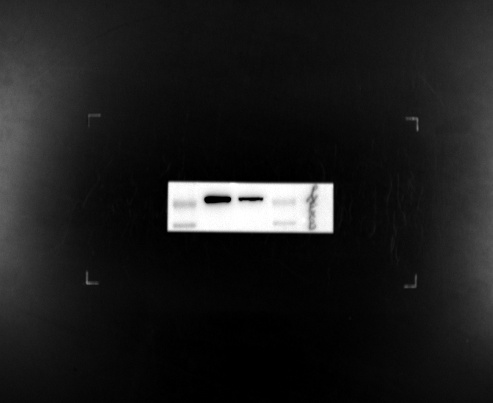

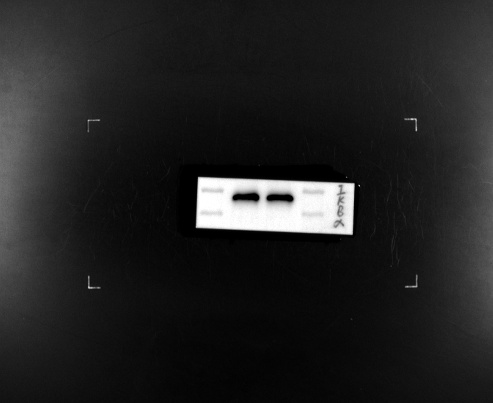


p-IkBα (PC-9) GAPDH (PC-9)


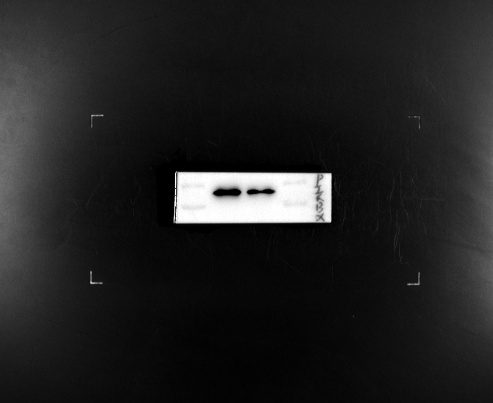

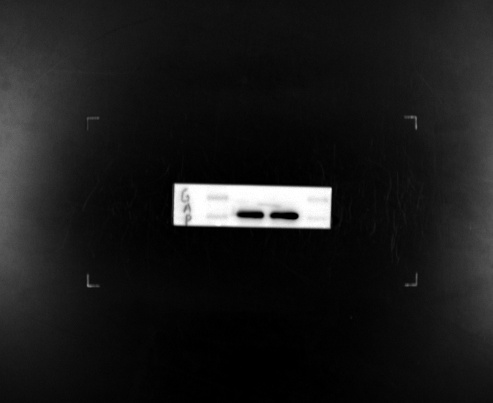


RECQL4 (NCI-H1299) NFκB (NCI-H1299)


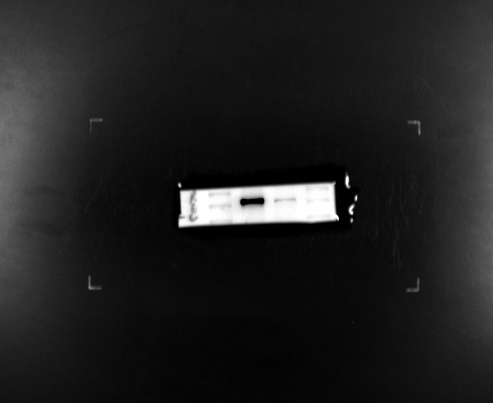

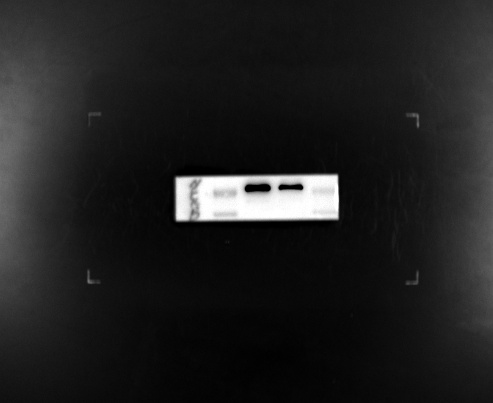


p-NFκB (NCI-H1299) IkBα (NCI-H1299)


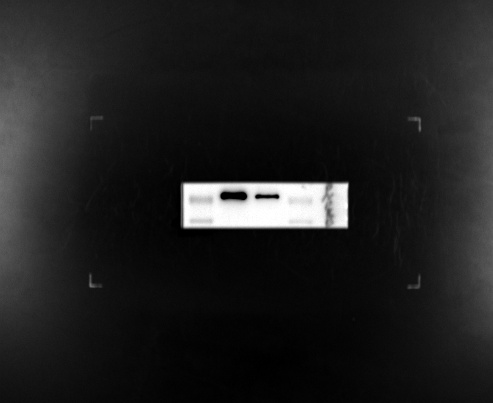

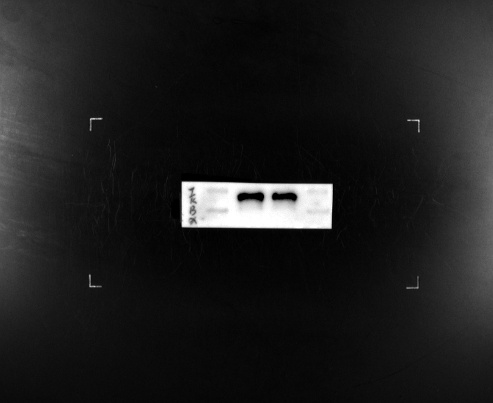


p-IkBα (NCI-H1299) GAPDH (NCI-H1299)


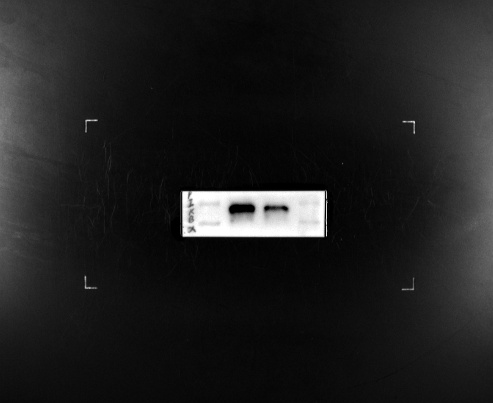

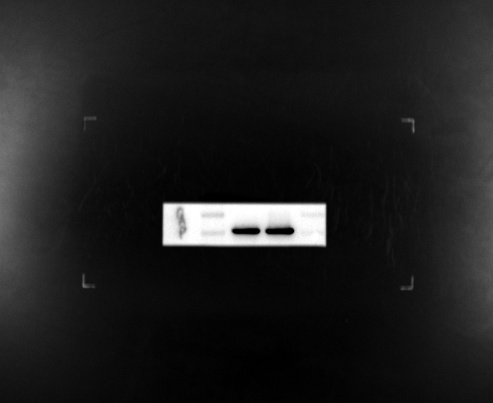


RECQL4 (A549) NFκB (A549)


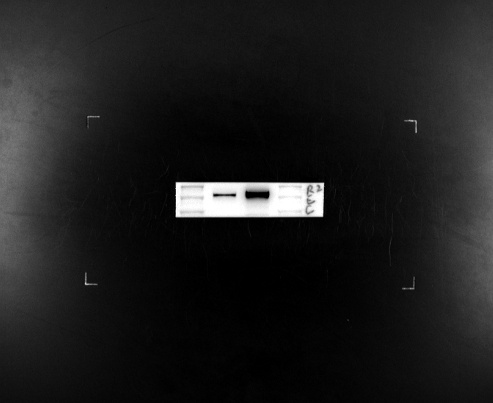

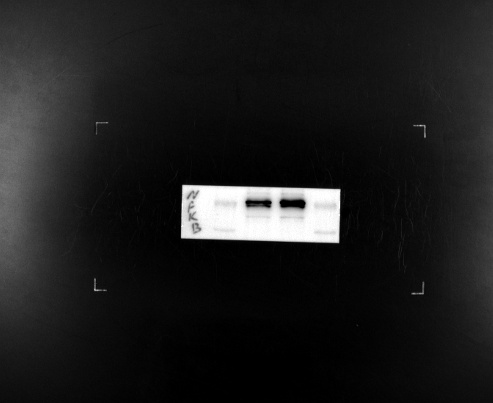


p-NFκB (A549) IkBα (A549)


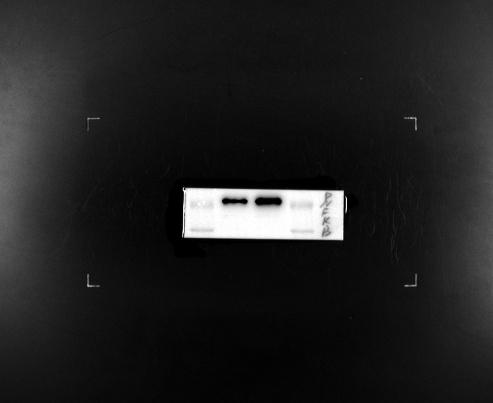

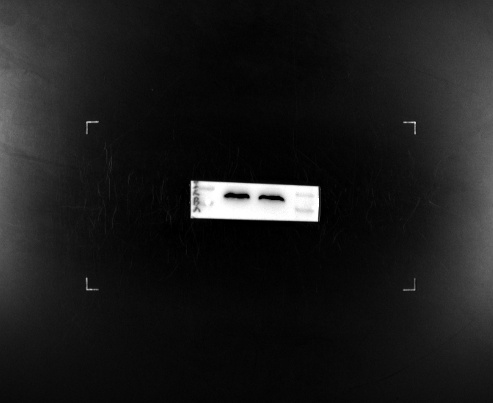


p-IkBα (A549) GAPDH (A549)


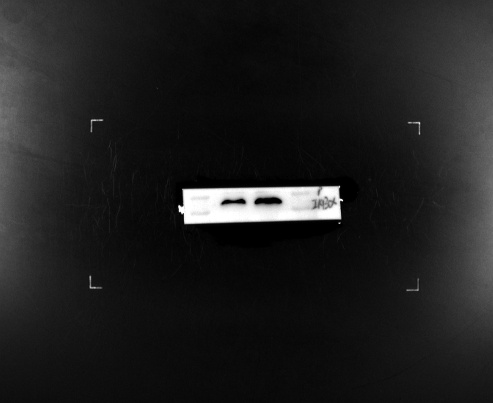

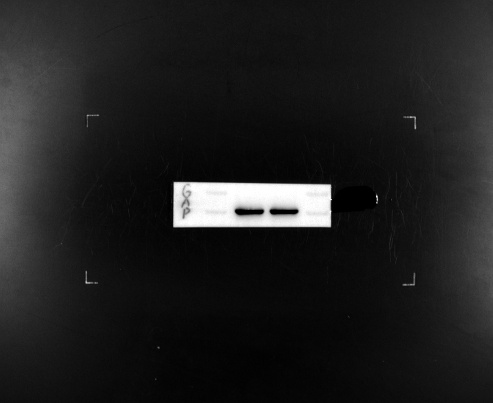


**Figure 6C** **(from top to bottom)**

A549 NCI-H1299

IP(RECQL4)+IB(RECQL4) IP(RECQL4)+IB(RECQL4)


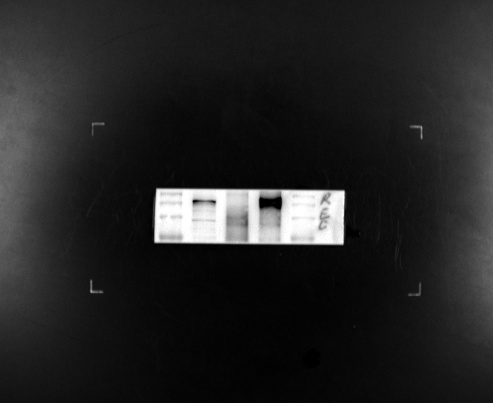

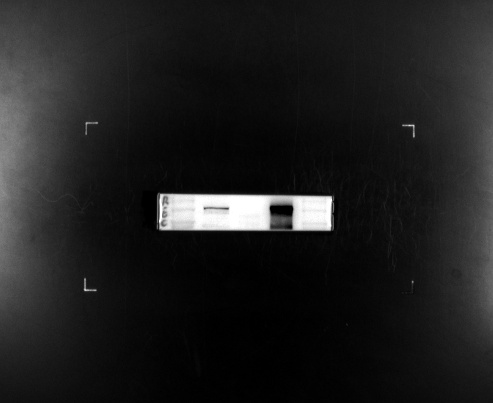


IP(RECQL4)+IB(G3BP1) IP(RECQL4)+IB(G3BP1)


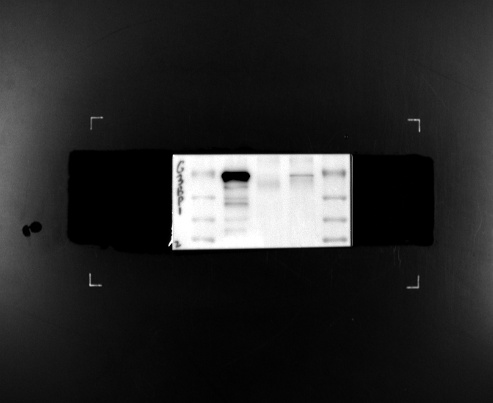

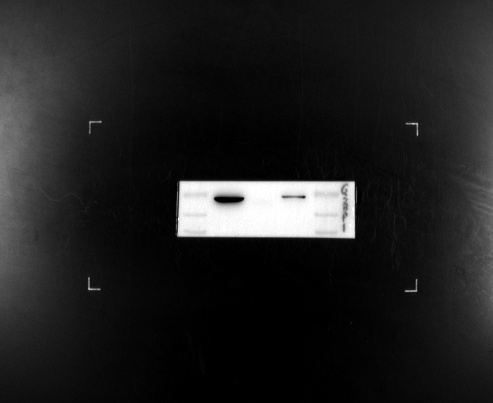


IP(RECQL4)+IB(YBX1) IP(RECQL4)+IB(YBX1)


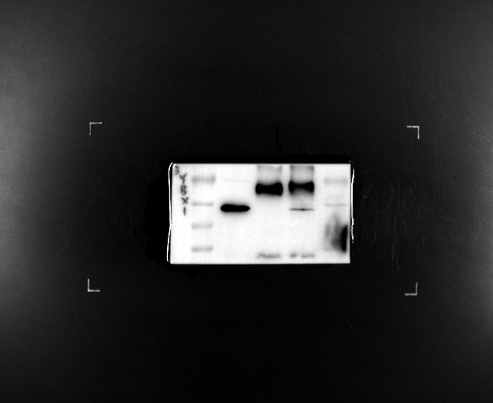

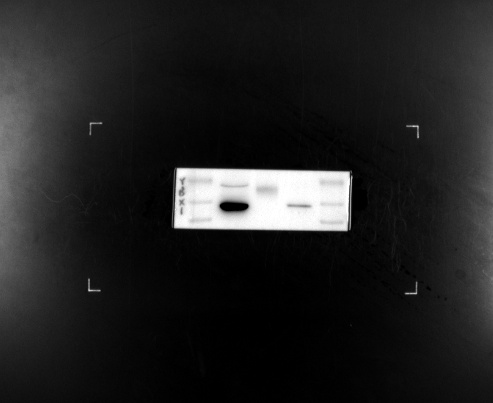


IP(YBX1)+IB(RECQL4) IP(YBX1)+IB(RECQL4)


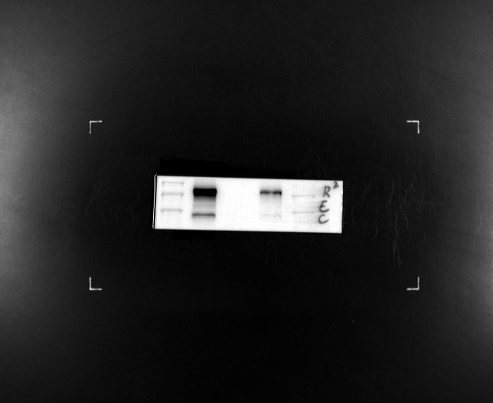

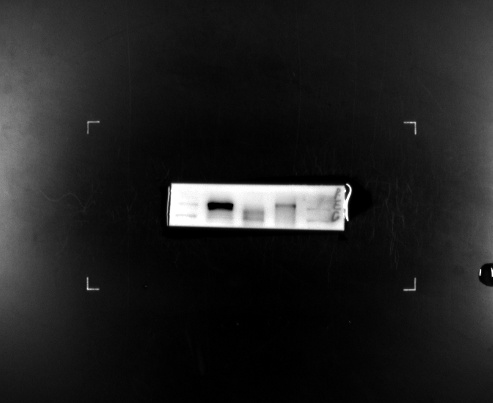


IP(YBX1)+IB(G3BP1) IP(YBX1)+IB(G3BP1)


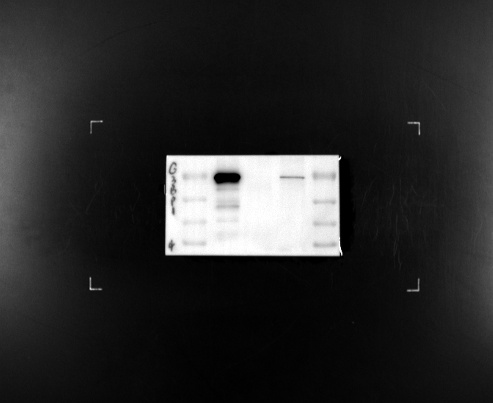

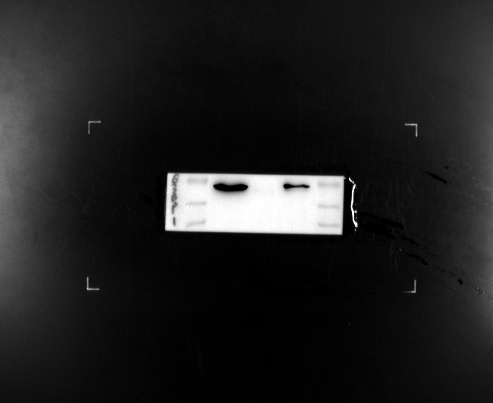


IP(YBX1)+IB(YBX1) IP(YBX1)+IB(YBX1)


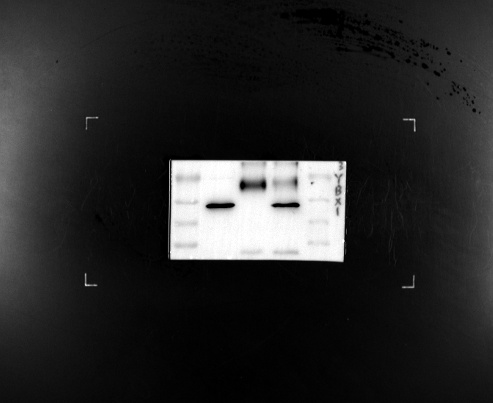

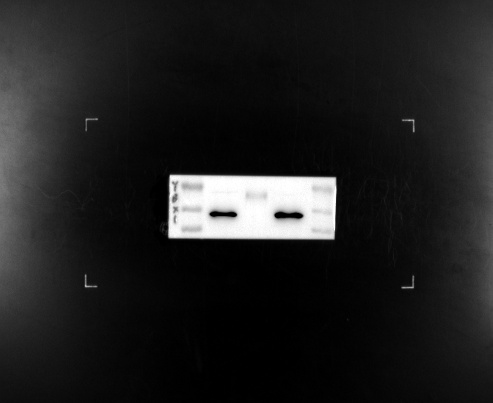


IP(G3BP1)+IB(RECQL4) IP(G3BP1)+IB(RECQL4)


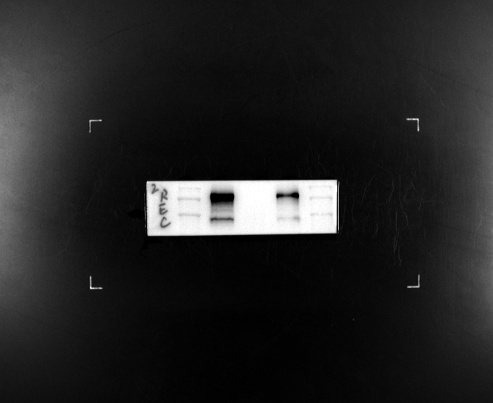

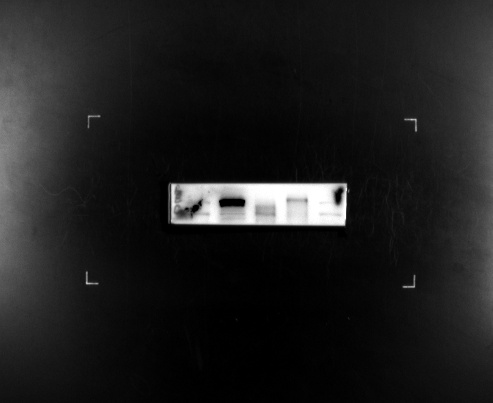


IP(G3BP1)+IB(G3BP1) IP(G3BP1)+IB(G3BP1)


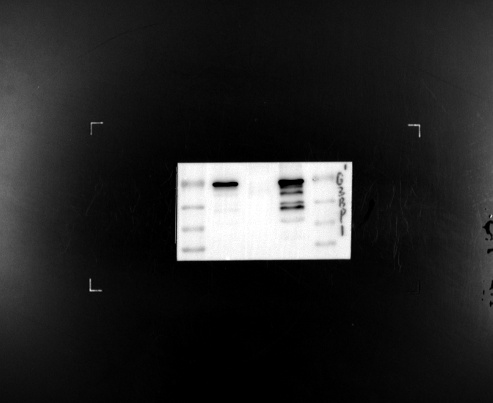

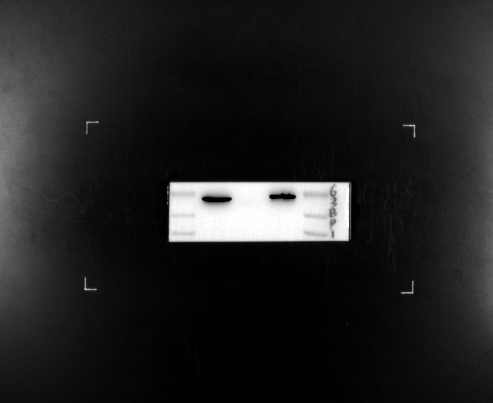


IP(G3BP1)+IB(YBX1) IP(G3BP1)+IB(YBX1)


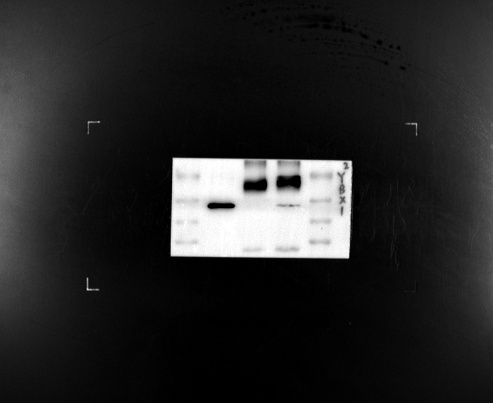

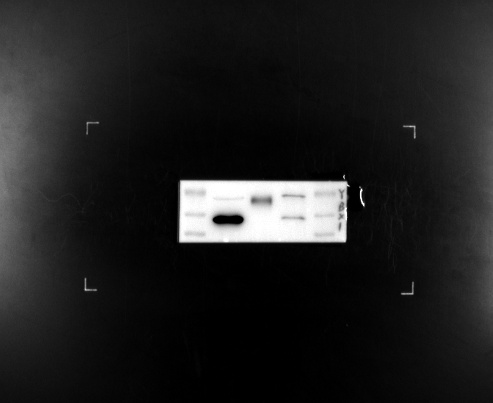


**Figure 6D (from top to bottom)**

IP(Flag)+IB(Flag) IP(Flag)+IB(Flag) IP(Myc)+IB(Myc)


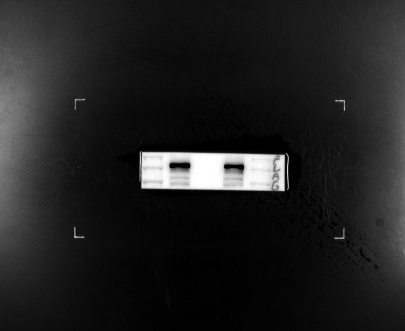

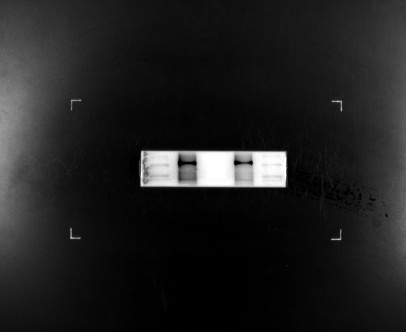

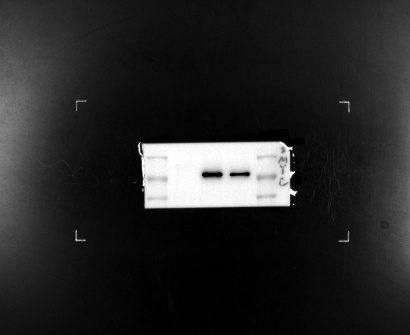


IP(Flag)+IB(HA) IP(Flag)+IB(Myc) IP(Myc)+IB(HA)


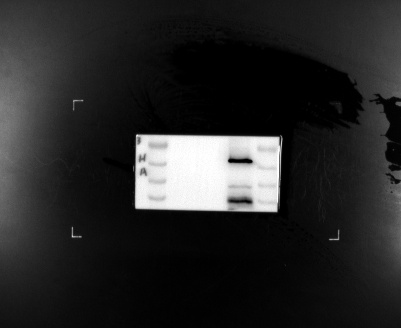

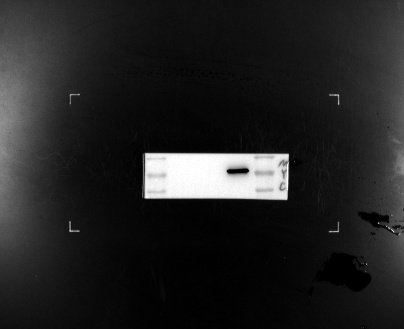

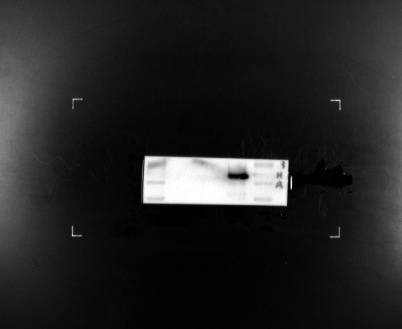


IP(HA)+IB(Flag) IP(Myc)+IB(Flag) IP(HA)+IB(Myc)


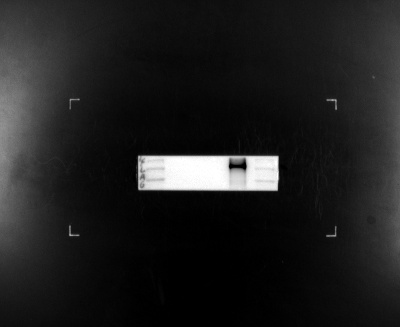

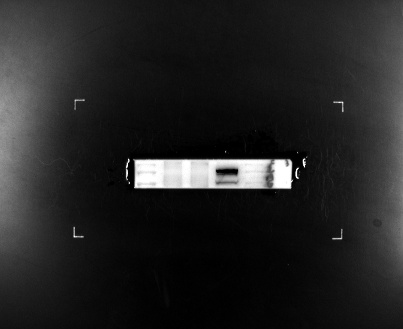

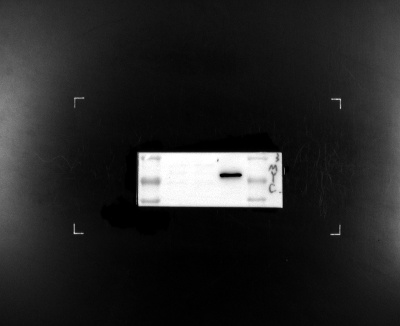


IP(HA)+IB(HA) IP(Myc)+IB(Myc) IP(HA)+IB(HA)


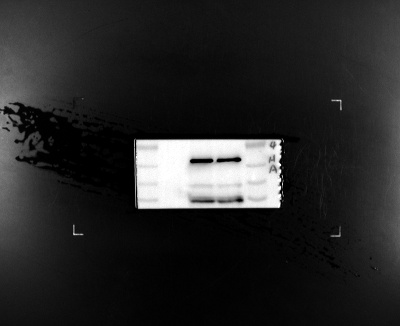

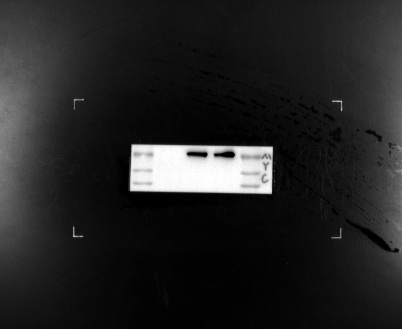

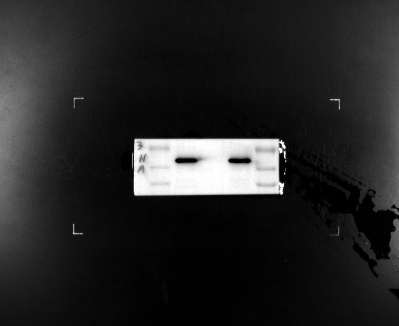


Input(Flag) Input(Flag) Input(Myc)


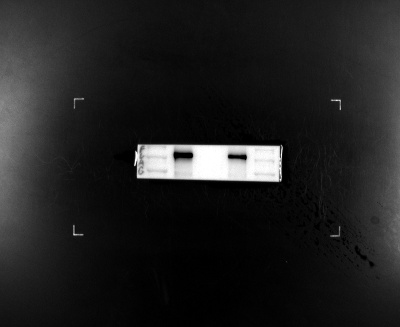

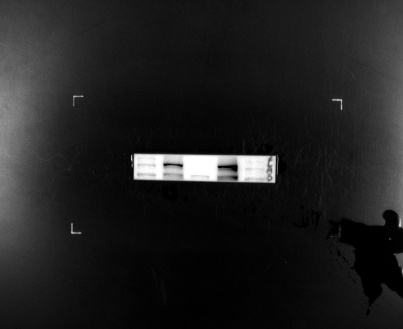

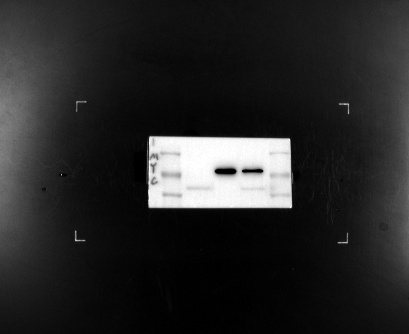


Input(HA) Input(Myc) Input(HA)


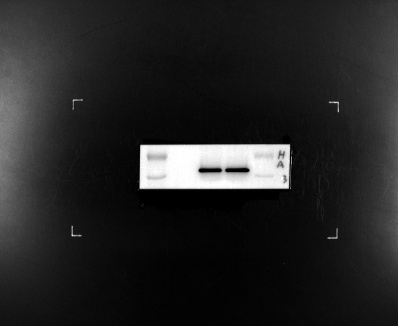

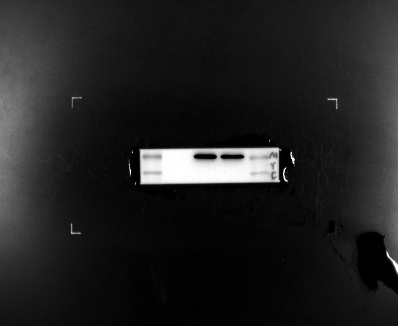

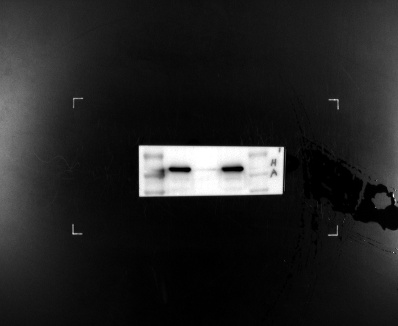


Input(GAPDH) Input(GAPDH) Input(GAPDH)


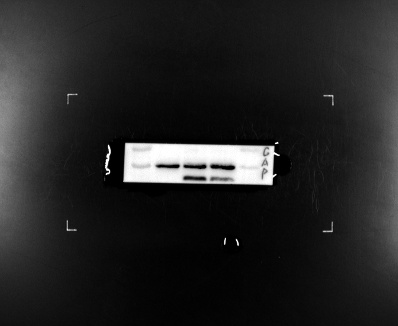

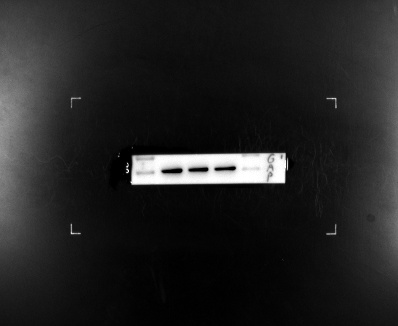

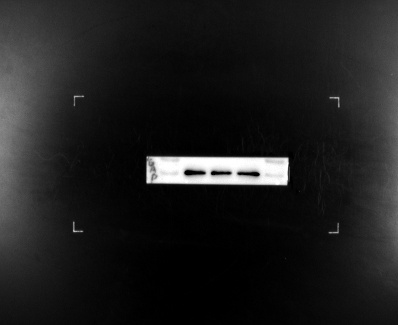


**Figure 6E (from top to bottom)**

A549 NCI-H1299

IP (IgG, YBX1, G3BP1) IP (IgG, YBX1, G3BP1)

IB (G3BP1) IB (G3BP1)


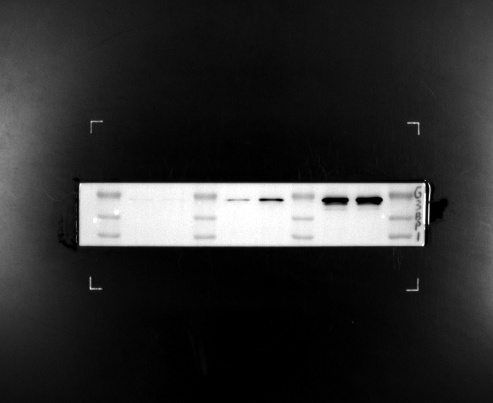

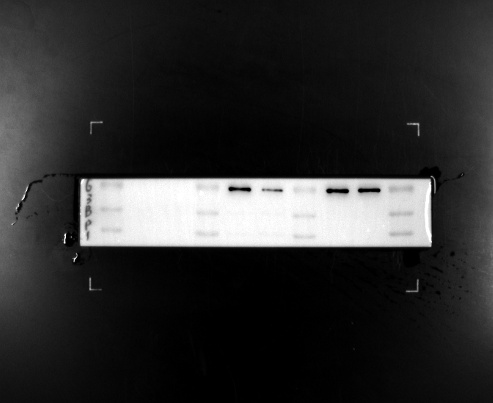


IP (IgG, YBX1, G3BP1) IP (IgG, YBX1, G3BP1)

IB (YBX1) IB (YBX1)

Input (RECQL4) Input (RECQL4)

Input (G3BP1) Input (G3BP1)

Input (YBX1) Input (YBX1)

Input (GAPDH) Input (GAPDH)

**Figure 6F (from top to bottom)**

IP(Myc)+IB(Myc)

IP(Myc)+IB(HA)

IP(HA)+IB(Myc)

IP(HA)+IB(HA)

Input (Flag)

Input (Myc)

Input (HA)

Input (GAPDH)

**Figure 7G**

RECQL4 G3BP1

YBX1 NFκB

p-NFκB IkBα

p-IkBα CCNE1

CCND1 CDK2

CDK4 GAPDH

**Supplementary Figure 3A (from top to bottom)**

RECQL4 (PC-9) RECQL4(NCI-H1299)

GAPDH (PC-9) GAPDH (NCI-H1299)

**Supplementary Figure 3F (from top to bottom)**

RECQL4 (PC-9) RECQL4(NCI-H1299)

CCNE1 (PC-9) CCNE1 (NCI-H1299)

CCND1 (PC-9) CCND1 (NCI-H1299)

CDK2 (PC-9) CDK2 (NCI-H1299)

CDK4 (PC-9) CDK4 (NCI-H1299)

GAPDH (PC-9) GAPDH (NCI-H1299)

**Supplementary Figure 4C (from top to bottom)**

RECQL4 (PC-9) RECQL4(NCI-H1299)

N-cadherin (PC-9) N-cadherin (NCI-H1299)

E-cadherin (PC-9) E-cadherin (NCI-H1299)

Vimentin (PC-9) Vimentin (NCI-H1299)

GAPDH (PC-9) GAPDH (NCI-H1299)

**Supplementary Figure 4D (from top to bottom)**

RECQL4 (PC-9) RECQL4(NCI-H1299)

NFκB (PC-9) NFκB (NCI-H1299)

p-NFκB (PC-9) p-NFκB (NCI-H1299)

IkBα (PC-9) IkBα (NCI-H1299)

p-IkBα (PC-9) p-IkBα (NCI-H1299)

GAPDH (PC-9) GAPDH (NCI-H1299)
